# Vaccine design via antigen reorientation

**DOI:** 10.1038/s41589-023-01529-6

**Published:** 2024-01-15

**Authors:** Duo Xu, Joshua J. Carter, Chunfeng Li, Ashley Utz, Payton A. B. Weidenbacher, Shaogeng Tang, Mrinmoy Sanyal, Bali Pulendran, Christopher O. Barnes, Peter S. Kim

**Affiliations:** 1 Department of Biochemistry, Stanford University School of Medicine, Stanford, CA USA; 2 Sarafan ChEM-H, Stanford University, Stanford, CA USA; 3 Stanford Biophysics Program, Stanford University School of Medicine, Stanford, CA USA; 4 Stanford Medical Scientist Training Program, Stanford University School of Medicine, Stanford, CA USA; 5 Institute for Immunity, Transplantation and Infection, Stanford University, Stanford, CA USA; 6 Department of Chemistry, Stanford University, Stanford, CA USA; 7 Department of Pathology, Stanford University School of Medicine, Stanford, CA USA; 8 Department of Microbiology and Immunology, Stanford University School of Medicine, Stanford, CA USA; 9 Department of Biology, Stanford University, Stanford, CA USA; 10 Chan Zuckerberg Biohub, San Francisco, CA USA

**Keywords:** Vaccines, Chemical modification, Infectious diseases, Peptides

## Abstract

A major challenge in creating universal influenza vaccines is to focus immune responses away from the immunodominant, variable head region of hemagglutinin (HA-head) and toward the evolutionarily conserved stem region (HA-stem). Here we introduce an approach to control antigen orientation via site-specific insertion of aspartate residues that facilitates antigen binding to alum. We demonstrate the generalizability of this approach with antigens from Ebola, severe acute respiratory syndrome coronavirus 2 (SARS-CoV-2) and influenza viruses and observe enhanced neutralizing antibody responses in all cases. We then reorient an H2 HA in an ‘upside-down’ configuration to increase the exposure and immunogenicity of HA-stem. The reoriented H2 HA (reoH2HA) on alum induced stem-directed antibodies that cross-react with both group 1 and group 2 influenza A subtypes. Electron microscopy polyclonal epitope mapping (EMPEM) revealed that reoH2HA (group 1) elicits cross-reactive antibodies targeting group 2 HA-stems. Our results highlight antigen reorientation as a generalizable approach for designing epitope-focused vaccines.

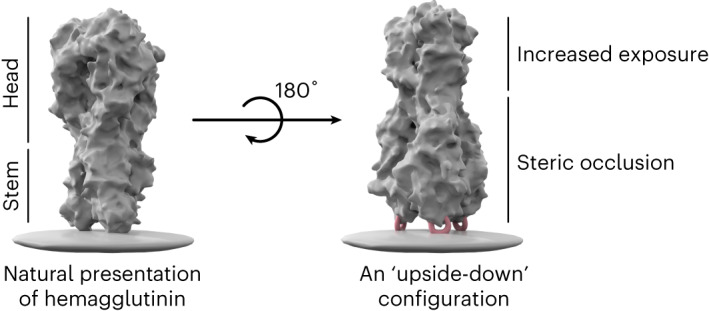

## Abstract (web summary)

A new design for vaccines consisting of reorienting the viral glycoprotein in an ‘upside-down’ configuration broadens immune responses to diverse influenza subtypes and serves as a proof of concept for designing a universal flu vaccine.

## Main

The rapid evolution of viruses such as influenza and severe acute respiratory syndrome coronavirus 2 (SARS-CoV-2) leads to periodic outbreaks with varying severity and represents a serious public health concern^1–3^. In the case of influenza virus, current vaccines elicit strain-specific antibody responses against the immunodominant but variable head region of HA (HA-head) that undergoes antigenic drift, necessitating annual updates of seasonal influenza vaccines^4,5^. When a novel zoonotic influenza virus acquires the ability of sustained human-to-human transmission, existing vaccine-induced immunity is unlikely to confer protection^6^. Such risks highlight the importance of pandemic preparedness and the urgent need to generate a universal vaccine that protects against diverse influenza strains^7^.

A potential strategy for developing more broadly protective vaccines is to focus the immune response (referred to as immunofocusing) on antigenic regions that are less likely to evolve. Because the stem region of HA (HA-stem) contains highly conserved epitopes across different influenza subtypes, focusing antibody responses on HA-stem and away from the immunodominant HA-head is a promising approach^8^. Human monoclonal antibodies (mAbs) targeting HA-stem show broad neutralizing activity and protect animals from lethal challenges^9–13^. HA-stem-directed antibodies also correlate with protection from infection and disease in humans^14^.

Current immunofocusing approaches include sequential immunization^15–18^ or mosaic display^19,20^ of heterologous HAs or chimeric HAs that contain the same HA-stem but varying HA-head to stimulate a stem-directed response^21^. These approaches require complicated vaccination regimens and do not directly address the immunodominance of HA-head^22^. Other immunofocusing approaches include masking HA-head with glycosylation^23,24^ or PEGylation^25^ or structure-based design of HA-stem-only immunogens^26–29^. While epitope masking often leads to reduced immunogenicity in vivo^24,25^, the generation of HA-stem-only proteins requires laborious rounds of design, screening and optimization of antigens to retain the proper presentation of conformational epitopes. Ultimately, although these strategies have found some success in inducing cross-reactivity among a particular phylogenetic group (that is, group 1 or group 2) of influenza A virus HAs, it remains a challenge to elicit a consistent response to both groups from a single immunogen.

In this study, we investigated the hypothesis that antigen reorientation is a straightforward and effective immunofocusing strategy. We tested if reorientation of HA in an ‘upside-down’ configuration from its natural presentation on influenza virions would sterically occlude HA-head and redirect the antibody response to the more exposed HA-stem. Interestingly, stem-directed (but not head-directed) mAbs isolated from vaccinated individuals exhibit significantly reduced binding to the whole virus as compared to recombinant HA proteins^30^. We hypothesize that steric occlusion leads to poor accessibility of HA-stem^31^, thereby biasing antibody responses toward HA-head during natural infection^30^.

To control antigen orientation, we first developed an approach based on the site-specific insertion of short stretches of aspartate residues (oligoD) into different protein antigens to facilitate their binding to alum, a well-established vaccine adjuvant that has been used for over 90 years^32^. The surface of alum allows the presentation of oligoD-modified antigens in a pre-defined manner. Methods to afford increased antigen binding to alum were developed previously^33–35^. Most notably, phosphoserine-tagged antigens showed increased binding to alum, leading to substantially enhanced humoral immune responses after immunization^35^. Still, this approach requires additional synthesis of phosphorylated peptides followed by chemical conjugation to antigens and post-modification purification procedures. In contrast, oligoD insertion is a simple, one-step process pre-programmed by molecular cloning. To validate our proposed approach, we inserted oligoD at the C-terminus of Ebola glycoprotein and showed that oligoD insertion increased antigen-binding to alum and elicited robust germinal center (GC) and neutralizing antibody responses. To demonstrate the simplicity and generalizability of our approach, we also inserted oligoD at two other protein antigens (SARS-CoV-2 spike and HA of A/New Caledonia/20/99) and found that oligoD-modified antigens induced significantly stronger neutralizing antibody responses compared to wild-type antigens.

After establishing that oligoD insertion mediated antigen binding to alum, we identified a permissive site on the head of an H2 HA (A/Japan/305/1957) for oligoD insertion. We chose this H2 HA because H2 HAs contain a bulky phenylalanine residue on their stem region that expands the breadth of B cell responses upon vaccination compared to HAs from other subtypes^36^. We showed that oligoD insertion into H2 HA resulted in the tri-valent anchoring of its head to alum at specific locations on each HA trimer in an ‘upside-down’ configuration. Immunization with this reoriented group 1 H2 HA (reoH2HA) on alum elicited a stem-directed and cross-reactive antibody response to diverse HA subtypes from both group 1 and group 2. Negative-stain electron microscopy polyclonal epitope mapping (nsEMPEM) also revealed that reoH2HA elicited cross-reactive antibodies that recognized stem epitopes of group 2 HAs, whereas H2 HA did not. Our results demonstrate the potential of designing epitope-focused vaccine candidates via antigen reorientation.

## Results

### OligoD insertion enables antigen binding to alum

**Fig. 1:**
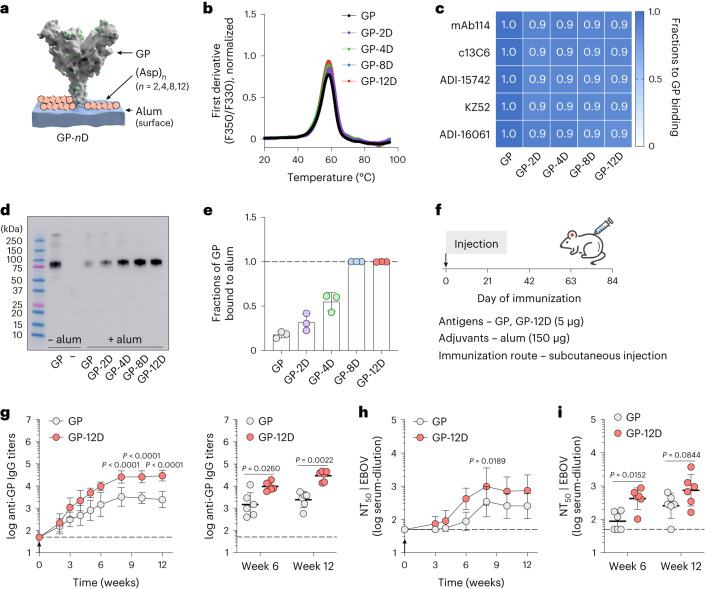
OligoD insertion enables antigen binding to alum and enhances neutralizing antibody responses. **a**, Site-specific insertion of oligoD of different lengths (2, 4, 8 or 12 aspartate residues, pink) at the C-terminus of Ebola GP (Protein Data Bank ID: 5JQ3). **b**, Thermal melting profiles of wild-type or oligoD-modified GP measured by differential scanning fluorimetry. **c**, Analysis of GP-specific mAb binding of wild-type or oligoD-modified GP by BLI. Shifts in nanometers of mAb-binding to oligoD-modified GP were normalized as a fraction of shifts to wild-type GP binding. Fractions were calculated based on data in Supplementary Fig. 1. **d**,**e**, Detection and quantification of alum-bound GP by western blot analysis (**d**) and ELISA (**e**). Upon separation, alum-bound GP was detected by mAb114 on the western blot, and unbound GP was quantified by ELISA as in Extended Data Fig. 1f. The dashed line indicates 100% binding to alum in **e**. Data are presented as mean ± s.d. (*n* = 3 samples per group). **f**, A single-dose immunization study with GP or GP-12D adjuvanted with alum via subcutaneous injection in BALB/c mice. **g**, Serum GP-specific IgG titers over time. Antibody titers at weeks 6 and 12 after immunization are plotted on the right for comparison of the two groups (*n* = 6 mice per group). **h**, Serum NT_50_ against EBOVs over time. **i**, NT_50_ of weeks 6 and 12 from the two groups are shown for comparison (*n* = 6 mice per group). Dashed lines indicate the limit of quantification in **g**–**i**. Data are presented as geometric mean ± s.d. of the log_10_‐transformed values (**g**–**i**). Comparison of IgG titers (**g**) or NT_50_ (**h**) over time was performed using two-way ANOVA followed by a Bonferroni test. Comparison of two groups was performed using the two-tailed Mann–Whitney *U*-test (**g**,**i**). *P* values of 0.05 or less were considered significant. Source data

**Extended Data Fig. 1:**
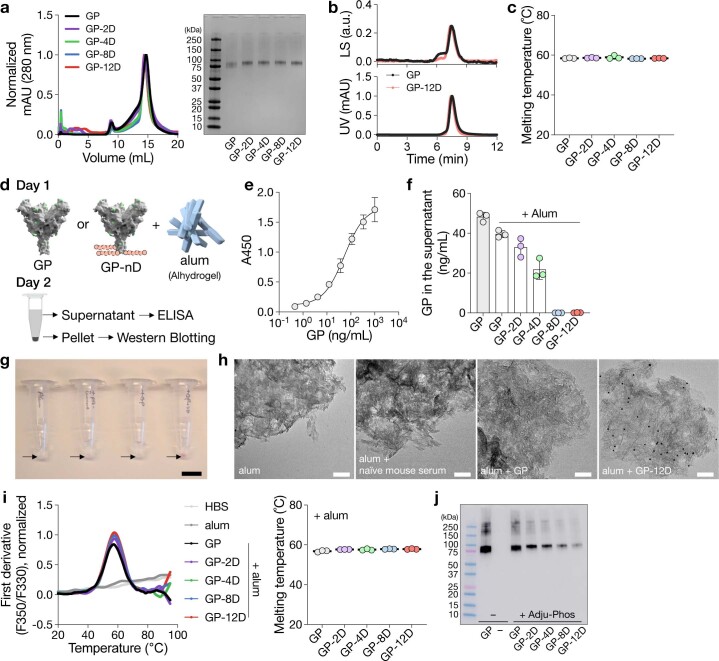
OligoD insertion into Ebola GP. **a**, Size-exclusion purification and gel electrophoresis of wild-type and oligoD-modified GP. Gel was stained with Coomassie brilliant blue. **b**, Size-exclusion chromatography coupled with multi-angle light scattering (SEC-MALS) analysis of GP and GP-12D on a Superdex 200 column. UV and LS indicate absorbance at 280 nm and light scattering signals, respectively. **c**, Thermal melting temperature (T_m_) of wild-type and oligoD-modified GP. Data are presented as mean ± s.d. (*n* = 3 samples per group). **d**, Alum-binding assay for wild-type or oligoD-modified GP. GP proteins were pre-mixed with alum for 1 h at room temperature and then incubated in PBS containing naïve mouse serum for 24 h at 37 °C. Upon centrifugation, the concentrations of unbound GP in the supernatant were quantified by ELISA. The rinsed pellet was analyzed by Western blotting to detect alum‐bound GP. **e**, Standard curve established with known concentrations of GP for reference. Data are presented as mean ± s.d. (*n* = 3 samples per group). **f**, Concentrations of unbound GP in the supernatant of GP-alum mixtures. Data are presented as mean ± s.d. (*n* = 3 samples per group). **g**, Immunogold labeling of alum complexed with GP or GP-12D. Alum alone and alum incubated with naïve mouse serum served as controls. Arrows indicate alum pellets after staining. Scale bar, 1 cm. **h**, Representative transmission electron micrographs of antigen-alum complexes prepared from **g**. Scale bar, 100 nm. **i**, Thermal melting profiles (left) and T_m_ (right) of wild-type or oligoD-modified GP in the presence of alum. Data are presented as mean ± s.d. (*n* = 3 samples per group). **j**, Detection of Adju-Phos-bound GP by Western-blot analysis. Adju-Phos was used instead of Alhydrogel in the antigen-binding assay. Source data

**Extended Data Fig. 2:**
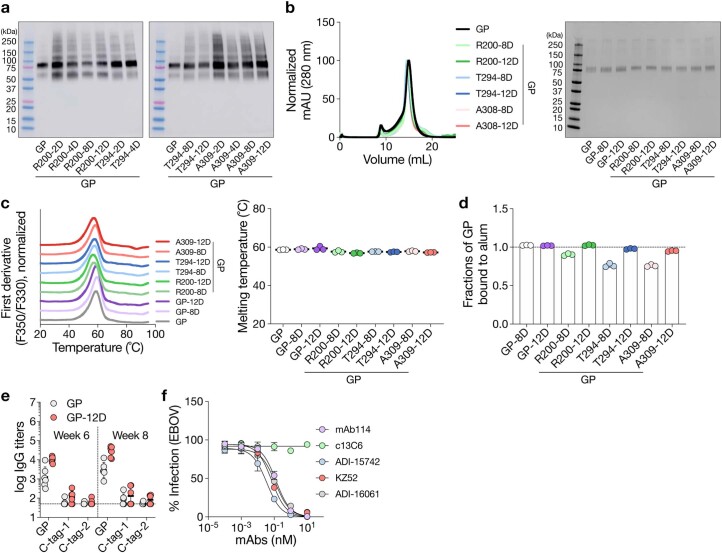
OligoD insertion into flexible loop regions on GP and immunogenicity of oligoD-modified GP in vivo. a, Screening of oligoD insertion into flexible loop regions on Ebola GP by Western-blot analysis. Insertions (2,4,8 or 12D) were made after residues R200, T294 or A309 as indicated. Supernatant from transient transfection was used for the analysis, and blots were detected by mAb114. b, Size-exclusion purification and gel electrophoresis of oligoD-modified GP after purification. Gel was stained with Coomassie brilliant blue. c, Thermal melting profiles and T_m_ of wild-type and oligoD-modified GP. Data are presented as mean ± s.d. in the dot plot (n = 3 samples per group). d, Quantification of the alum-bound fraction of oligoD-modified GP by ELISA (n = 3 samples per group). The dashed line indicates 100% binding to alum. e, Serum binding titers of GP or C-terminal tags (C-Tag-1 = ZsGreen-Avi-His-12D; C-Tag-2 = GFP-GCN4-His) by ELISA. The dashed line indicates the limit of quantification. Data are presented as the geometric mean ± s.d. of the log_10_‐transformed values (n = 6 mice per group). f, Validation of the neutralization assay against Ebola GP-pseudotyped lentiviruses (EBOVs) with the five GP-specific mAbs. c13C6 is known to be non-neutralizing and serves as a control. Data are presented as mean ± s.d. (n = 4 technical replicates). Source data

To determine the length of oligoD required for alum binding, we first used the ectodomain of Ebola glycoprotein (GP) as a test case and genetically inserted 2, 4, 8 or 12 repeating units of aspartates (abbreviated as 2D, 4D, 8D or 12D, respectively) at its C-terminus (Fig. 1a). OligoD-modified GP proteins were expressed in Expi293F cells via transient transfection and purified to homogeneity (Extended Data Fig. 1a,b). OligoD insertion did not change the thermal melting profile (Fig. 1b) or melting temperature (T_m_, 58 °C; Extended Data Fig. 1c) of GP, suggesting a negligible perturbation to its native structure. In addition, we selected a panel of five GP-specific mAbs (mAb114, c13C6, ADI-15742, KZ52 and ADI-16061) that recognize different epitopes on GP and examined their binding to wild-type or oligoD-modified GP using bio-layer interferometry (BLI) (Supplementary Fig. 1). We found no noticeable differences in the binding profiles of these mAbs to all GP proteins (Fig. 1c), suggesting proper presentation of those conformational epitopes. Next, we measured the effect of oligoD insertions on GP-binding to alum (Alhydrogel). We pre-mixed wild-type or oligoD-modified GP with alum (protein:alum, 1:10, w/w) for 1 h and then incubated the mixtures in the presence of naive mouse serum (10%, v/v) at 37 °C for 24 h (Extended Data Fig. 1d). After incubation, alum pellets were rinsed extensively and subjected to protein gel electrophoresis followed by western blot analysis to determine the amount of alum-bound GP. Whereas only a small fraction of wild-type GP was detected on the blot, an increasing amount of GP bound to alum as the number of aspartates increased from two to 12 (Fig. 1d). To quantify the amount of alum-bound GP, we collected the supernatant from GP–alum mixtures and measured the concentration of unbound GP by ELISAs (Extended Data Fig. 1e). Consistent with the western blot, higher GP binding to alum correlated with increasing oligoD length (Fig. 1e and Extended Data Fig. 1f). The presence of naive mouse serum did not affect binding of GP-8D or GP-12D to alum. To directly visualize antigen binding to alum, we performed immunogold labeling for GP–alum complexes (Extended Data Fig. 1g) and observed the absorption of GP-12D (but not GP) on alum under transmission electron microscopy (Extended Data Fig. 1h). The interaction between oligoD and alum also did not affect the thermal stability of GP proteins (Extended Data Fig. 1i). Because oligoD likely mediates its alum binding via electrostatic interactions, use of the negatively charged aluminum phosphate (Adju-Phos) adjuvant abolished antigen binding as the length of oligoD increased (Extended Data Fig. 1j). Besides the C-terminus, we identified three flexible loop regions (after residue R200, T294 or A309) on GP that readily accommodated oligoD insertion (Extended Data Fig. 2a,b). Thermal melting profiles and T_m_ of the resulting oligoD-modified GP proteins were identical to those of wild-type GP (Extended Data Fig. 2c). Although both GP-8D and GP-12D exhibited approximately 100% binding to alum, only 12D insertion afforded approximately 100% GP binding to alum when inserted into each of these flexible loop regions (Extended Data Fig. 2d). Therefore, we inserted 12D into antigens to promote maximal binding to alum for subsequent immunization studies.

To evaluate the immunogenicity of oligoD-modified GP, we immunized mice with GP or GP-12D adjuvanted with alum via subcutaneous injection (Fig. 1f). A single dose of GP with alum elicited a relatively weak GP-specific antibody response, whereas GP-12D with alum stimulated a much stronger response with almost 10-fold higher IgG titers 6 weeks after immunization (Fig. 1g). At 6 weeks and 8 weeks after immunization, we detected very low IgG titers against oligoD or other C-terminal affinity tags that were used for purification purposes (Extended Data Fig. 2e). Next, we generated Ebola GP-pseudotyped lentiviruses (EBOVs) (Extended Data Fig. 2f) to analyze the neutralizing antibody response. Consistent with the IgG titers, GP-12D induced a three-fold to five-fold stronger neutralizing antibody response than GP did (Fig. 1h). Whereas all mice immunized with GP-12D adjuvanted with alum developed neutralizing responses at week 6 (Supplementary Fig. 2a), only half of the mice immunized with GP did, although the titers were lower. Even at week 12, one mouse immunized with GP did not show any neutralizing activity (Fig. 1i and Supplementary Fig. 2b). Taken together, oligoD insertion increased antigen binding to alum and significantly enhanced neutralizing antibody responses in animal immunizations compared to wild-type GP.

### OligoD-modified antigens stimulate robust GC responses

**Fig. 2:**
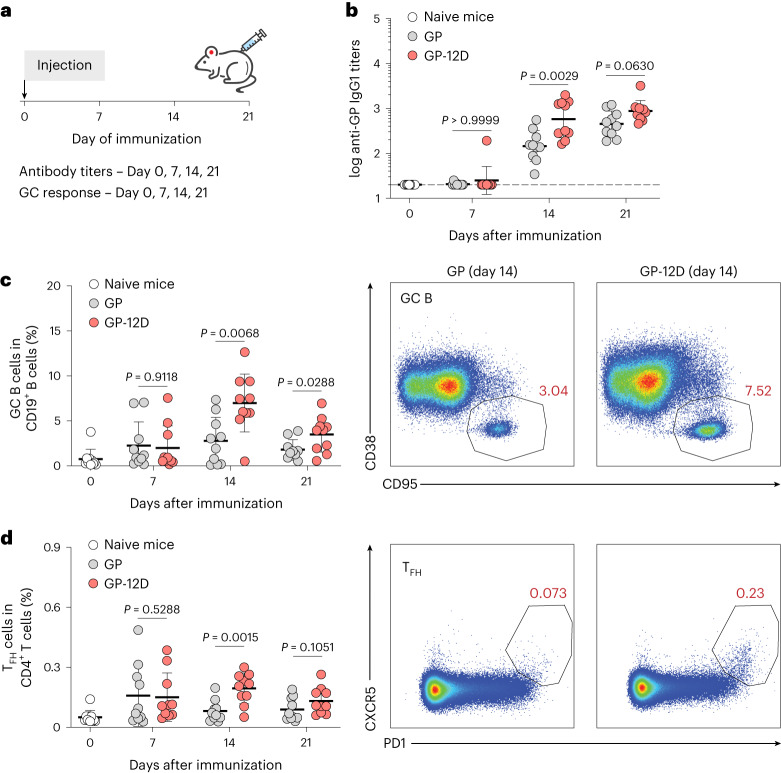
OligoD-modified GP stimulates a robust GC response. **a**, Immunization schedule for the analysis of GC responses in draining lymph nodes. BALB/c mice were immunized with GP or GP-12D adjuvanted with alum. Antibody titers and GC responses were measured before immunization and 7 d, 14 d or 21 d after immunization. **b**, Serum GP-specific IgG1 titers over time (*n* = 10 mice per antigen per timepoint). Data are presented as geometric mean ± s.d. of the log_10_‐transformed values. Dashed lines indicate the limit of quantification. **c**,**d**, Analysis of GC B cell (**c**) and T_FH_ cell (**d**) responses after immunization. Each circle represents a single mouse (*n* = 10 mice per antigen per timepoint). Data are presented as mean ± s.d. Comparison of two groups was performed using the two-tailed Mann–Whitney *U*-test (**b**–**d**). *P* values of 0.05 or less were considered significant. Source data

**Extended Data Fig. 3:**
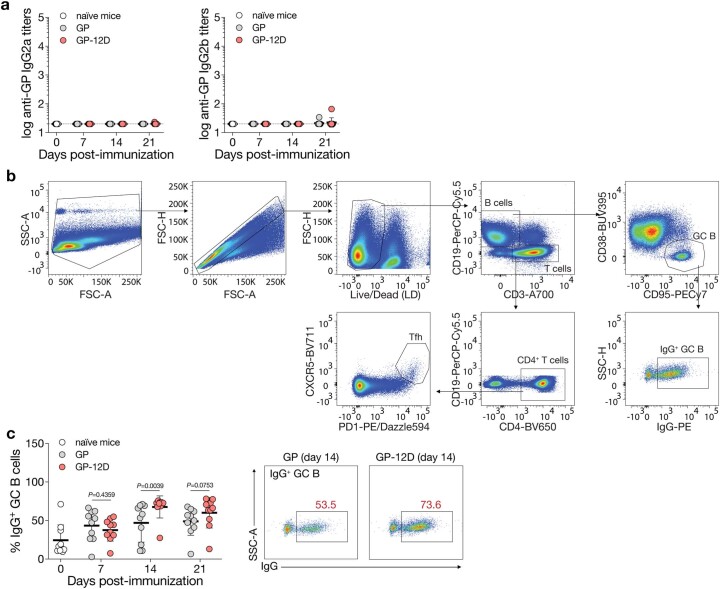
Analysis of germinal center responses. a, Serum GP-specific IgG2a or IgG2b titers 7 d, 14 d or 21 d post-immunization (n = 10 mice per antigen per time point). Dashed lines indicate the limit of quantification. Data are presented as the geometric mean ± s.d. of the log_10_‐transformed values. b, Gating strategy for the analysis of germinal center B cells (CD19^+^CD95^+^CD38^−^), IgG^+^ germinal center B cells and T follicular helper cells (CD3^+^CD4^+^PD1^+^CXCR5^+^) in the draining lymph nodes. c, Analysis of IgG^+^ GC B cell responses after immunization. Each circle represents a single mouse (n = 10). Data are presented as mean ± s.d. Comparison of two groups was performed using the two-tailed Mann–Whitney *U*-test. *P* values of 0.05 or less were considered significant. Source data

To understand the mechanism underlying the enhanced antibody response elicited by GP-12D, we immunized mice with GP or GP-12D and analyzed the GC responses in draining lymph nodes at 7 d, 14 d or 21 d after immunization using flow cytometry^37^ (Fig. 2a). As with the previous immunization (Fig. 1g), a single injection of GP-12D with alum elicited a stronger antibody response than GP with alum did (Fig. 2b). Use of alum also led to a Th2-biased response (IgG1) in both groups with negligible titers of IgG2a or IgG2b (Extended Data Fig. 3a). Besides the serological response, we examined the responses of GC B cells (CD19^+^CD95^+^CD38^−^), IgG^+^ GC B cells and T follicular helper cells (T_FH_, CD3^+^CD4^+^PD1^+^CXCR5^+^) in the draining lymph nodes (Extended Data Fig. 3b). Compared to GP with alum, GP-12D with alum induced an extended GC response with a substantially higher magnitude of GC B cells (Fig. 2c), IgG^+^ GC B cells (Extended Data Fig. 3c) and T_FH_ cells (Fig. 2d), which all peaked at 14 d after immunization. These data suggest that the higher antibody titers elicited by GP-12D with alum resulted from stimulation of a robust GC response.

### OligoD insertion is generalizable to other protein antigens

**Fig. 3:**
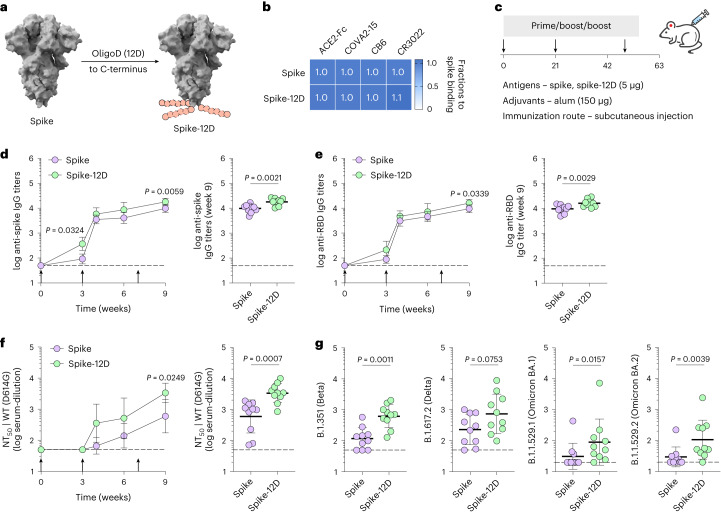
OligoD insertion into spike enhances neutralizing antibody responses. **a**, Insertion of oligoD (12D) at the C-terminus of SARS-CoV-2 spike (Protein Data Bank ID: 6VXX). **b**, BLI binding analysis of spike or spike-12D with ACE2–Fc and three spike-specific mAbs. **c**, A three-dose immunization study with spike or spike-12D adjuvanted with alum via subcutaneous injection in BALB/c mice. **d**,**e**, Serum spike-specific (**d**) or RBD-specific (**e**) IgG titers over time (*n* = 10 mice per group). Endpoint (week 9) antibody titers are plotted on the right for comparison of the two groups. **f**, Serum NT_50_ against SARS-CoV-2 spike-pseudotyped lentiviruses over time (*n* = 10 mice per group). Endpoint (week 9) NT_50_ are plotted on the right for comparison of the two groups. **g**, Endpoint (week 9) NT_50_ against SARS-CoV-2 variants of concern from the two groups. Each circle represents a single mouse (*n* = 10 mice per group). Dashed lines indicate the limit of quantification in **d**–**g**. Arrows in **d**–**f** indicate prime and boost immunizations. Data are presented as geometric mean ± s.d. of the log_10_‐transformed values. Comparison of IgG titers (**d**,**e**) or NT_50_ (**f**) over time was performed using two-way ANOVA followed by a Bonferroni test. Comparison of two groups was performed using the two-tailed Mann–Whitney *U*-test (**d**–**g**). *P* values of 0.05 or less were considered significant. Source data

**Extended Data Fig. 4:**
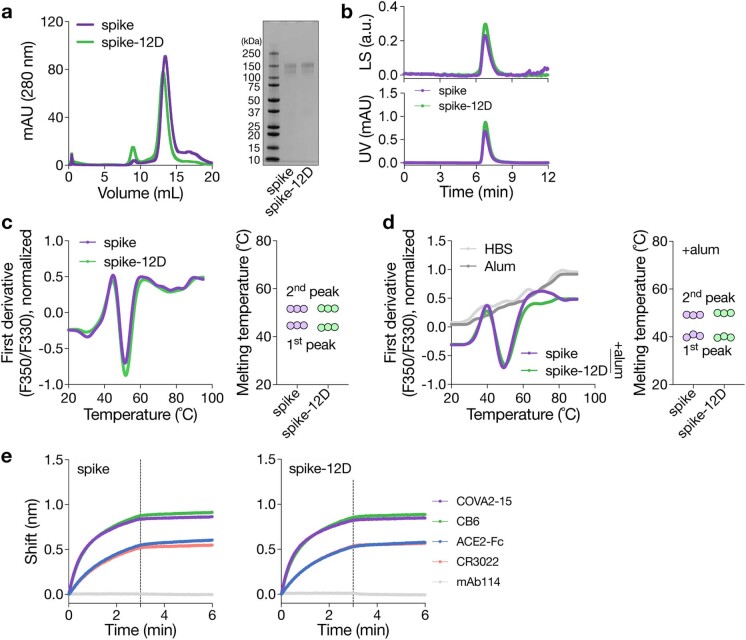
OligoD insertion into SARS-CoV-2 spike. a, Size-exclusion purification and gel electrophoresis of wild-type or oligoD-modified spike. Gel was stained with Coomassie brilliant blue. b, SEC-MALS analysis of spike and spike-12D on a Superdex 200 column. UV and LS indicate absorbance at 280 nm and light scattering signals, respectively. c,d, Thermal melting profiles and T_m_ of wild-type and oligoD-modified spike in the absence (c) or presence (d) of alum (spike: alum = 1:10, w/w in d) (n = 3 samples per group). e, BLI binding profiles of wild-type or oligoD-modified spike with ACE2-Fc and three Spike-specific mAbs (COVA2-15, CB6 and CR3022). A GP-specific mAb (mAb114) served as a negative control. Vertical dashed lines indicate the beginning of dissociation. Source data

**Extended Data Fig. 5:**
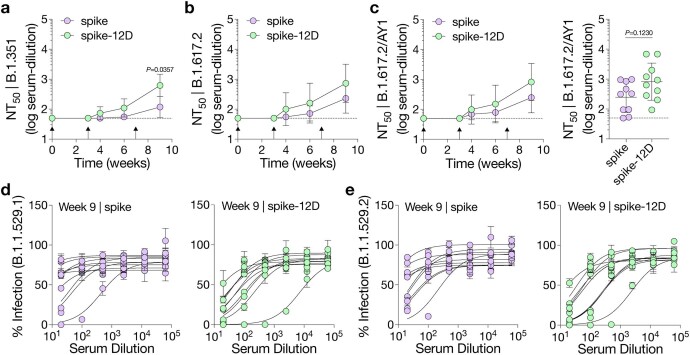
Neutralization of SARS-CoV-2 pseudoviruses. a-c, Serum neutralization titers (NT_50_) of SARS-CoV-2 variants of concern over time, including B.1.351 (a), B.1.617.2 (b) and B.1.617.2/AY1 (c) (n = 10 mice per group). Data are presented as the geometric mean ± s.d. of the log_10_‐transformed values in NT_50_. Dashed lines indicate the limit of quantification of NT_50_. Comparison of NT_50_ over time was performed using two-way ANOVA followed by a Bonferroni test. Comparison of two groups was performed using the two-tailed Mann–Whitney *U*-test. *P* values of 0.05 or less were considered significant. d,e, Serum neutralization profiles of Omicron variants B.1.1.529.1 (d) and B.1.1.529.2 (e). Each curve is derived from a single mouse (n = 10). Data are presented as mean ± s.d. of technical duplicates in neutralization curves. Source data

Given the simplicity of our approach, we applied oligoD insertion to two other protein antigens for additional validation. We first inserted oligoD at the C-terminus of SARS-CoV-2 spike to generate spike-12D (Fig. 3a). Wild-type spike and spike-12D were transiently expressed and purified to homogeneity (Extended Data Fig. 4a,b). In both the absence (Extended Data Fig. 4c) and the presence (Extended Data Fig. 4d) of alum, spike-12D shared the same thermal melting profile and T_m_ transitions (44 °C and 52 °C) with spike. We also found nearly identical binding profiles for ACE2–Fc and three spike-specific mAbs (COVA2-15, CB6 and CR3022) against spike or spike-12D (Fig. 3b and Extended Data Fig. 4e). Next, we immunized mice with spike or spike-12D adjuvanted with alum via subcutaneous injection (Fig. 3c). A three-dose regimen of spike-12D with alum elicited a significantly higher antibody response than spike with alum did against both spike (Fig. 3d) and its receptor-binding domain (RBD) (Fig. 3e). Immunization of spike-12D with alum also greatly enhanced the neutralizing response against spike-pseudotyped lentiviruses (Fig. 3f and Supplementary Fig. 3a,b). Despite the less than two-fold difference in IgG titers at week 9, the neutralization titers (NT_50_) of the spike-12D group were over five-fold higher than those of the spike group. When we further assessed the neutralization of other SARS-CoV-2 variants of concern (Extended Data Fig. 5a–e and Supplementary Fig. 3c–e), we found consistently higher neutralizing activity from antisera elicited by spike-12D (Fig. 3g).

**Fig. 4:**
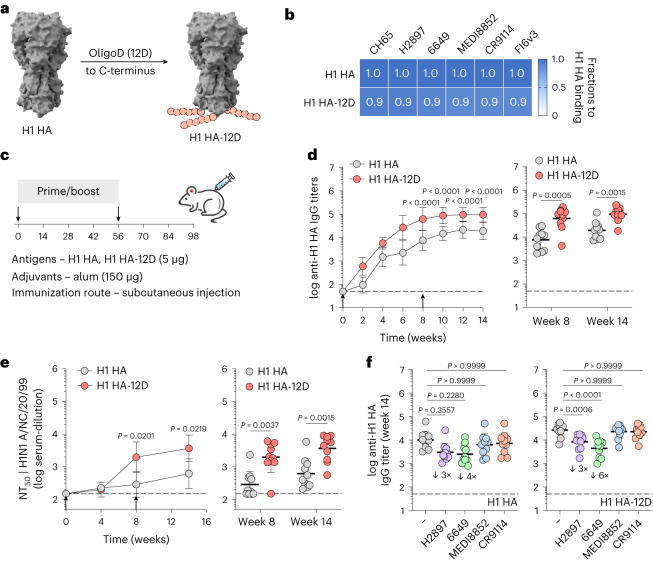
OligoD insertion into HA enhances neutralizing antibody responses. **a**, Insertion of oligoD (12D) at the C-terminus of H1 HA (Protein Data Bank ID: 1RU7 for reference). **b**, BLI binding analysis of H1 HA or H1 HA-12D with HA-specific mAbs. **c**, A prime-boost immunization study with H1 HA or H1 HA-12D adjuvanted with alum via subcutaneous injection in BALB/c mice. **d**, Serum H1 HA-specific IgG titers over time (*n* = 10 mice per group). Antibody titers of week 8 and week 14 from the two groups are plotted on the right for comparison (each circle represents a single mouse). **e**, Serum NT_50_ against authentic H1N1 A/NC/20/99 viruses over time (*n* = 10 mice per group). NT_50_ at weeks 8 and 14 are plotted on the right for comparison (each circle represents a single mouse). **f**, Serum binding titers to H1 HA in the presence of competing mAbs. Each circle represents a single mouse. Fold change is indicated by arrows with numbers. Dashed lines indicate the limit of quantification in **d**–**f**. Arrows in **d**,**e** indicate prime and boost immunizations. Data are presented as geometric mean ± s.d. of the log_10_‐transformed values. Comparison of IgG titers (**d**) or NT_50_ (**e**) over time was performed using two-way ANOVA followed by a Bonferroni test. Comparison of two groups was performed using the two-tailed Mann–Whitney *U*-test (**d**,**e**). Comparison of multiple groups was performed using one-way ANOVA followed by a Bonferroni test (**f**). *P* values of 0.05 or less were considered significant. Source data

**Extended Data Fig. 6:**
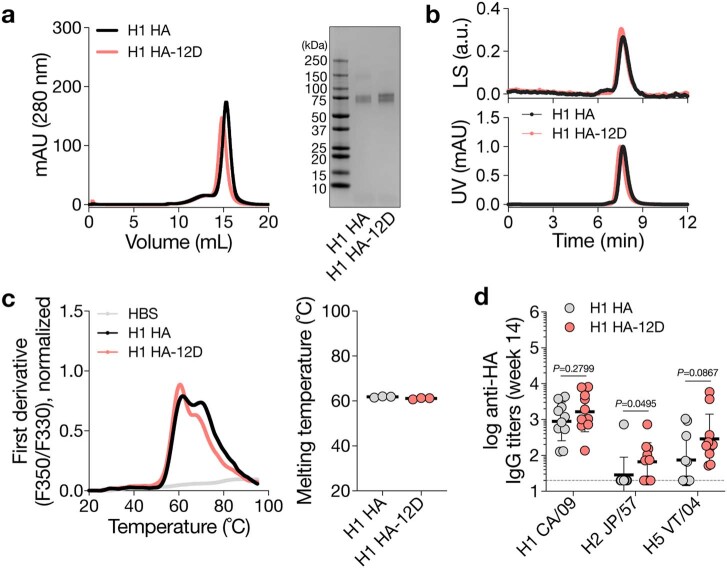
OligoD insertion into H1 HA. a, Size-exclusion purification and gel electrophoresis of wild-type or oligoD-modified H1 HA. Gel was stained with Coomassie brilliant blue. b, SEC-MALS analysis of H1 HA and H1 HA-12D on a Superdex 200 column. UV and LS indicate absorbance at 280 nm and light scattering signals, respectively. c, Thermal melting profiles and T_m_ of wild-type and oligoD-modified H1 HA. Data are presented as mean ± s.d. (n = 3 samples per group). d, Serum binding titers to different group 1 HAs (H1 CA/09 – A/California/07/2009, H2 JP/57 – A/Japan/305/1957, H5 VT/04 – A/Vietnam/1203/2004) (n = 10 mice per group). Each circle represents a single mouse (n = 10 mice per group). Dashed lines indicate the limit of quantification. Data are presented as the geometric mean ± s.d. of the log_10_-transformed values. Comparison of two groups was performed using the two-tailed Mann–Whitney *U*-test. *P* values of 0.05 or less were considered significant. Source data

Besides SARS-CoV-2 spike, we inserted oligoD at the C-terminus of an H1 HA (A/New Caledonia/20/99) (Fig. 4a). Similar to the modifications in GP and spike, oligoD insertion did not affect the expression, homogeneity (Extended Data Fig. 6a,b) or thermal stability (Extended Data Fig. 6c) of H1 HA. The binding profiles with different HA-specific mAbs (CH65, H2897, 6649, MEDI8852, CR9114 and FI6v3), as determined by BLI, also suggested the proper presentation of conformational epitopes on H1 HA-12D (Supplementary Fig. 4a and Fig. 4b). A prime-boost immunization of H1 HA-12D with alum elicited substantially higher (five-fold to 10-fold) HA-specific IgG titers than H1 HA with alum did (Fig. 4c,d). Increased IgG titers also translated into more potent neutralizing activity (Fig. 4e), where H1 HA-12D induced significantly higher NT_50_ than H1 HA did against authentic A/New Caledonia/20/1999 (H1N1 A/NC/20/99) viruses (Supplementary Fig. 4b,c). To determine whether the antisera targeted HA-head or HA-stem, we performed competition ELISA, in which antigen-coated ELISA plates were pre-incubated with a saturating concentration of individual HA-specific mAbs before incubation with antisera. We observed a clear competition between antisera and HA-head-directed mAbs (H2897 or 6649) with a more pronounced change in the H1 HA-12D group (Fig. 4f). In contrast, there was negligible competition with HA-stem-directed antibodies (MEDI8852 or CR9114), suggesting that oligoD insertion at the C-terminus of HA only boosted antibody responses toward the immunodominant HA-head. Nonetheless, higher antibody titers elicited by H1 HA-12D correlated with an increased breadth against two other group 1 HAs, namely H2 (A/Japan/305/1957) and H5 (A/Vietnam/1203/2004) HAs, presumably toward conserved epitopes on HA-head (Extended Data Fig. 6d).

### Reorientation of H2 HA on alum to afford immunofocusing

**Fig. 5:**
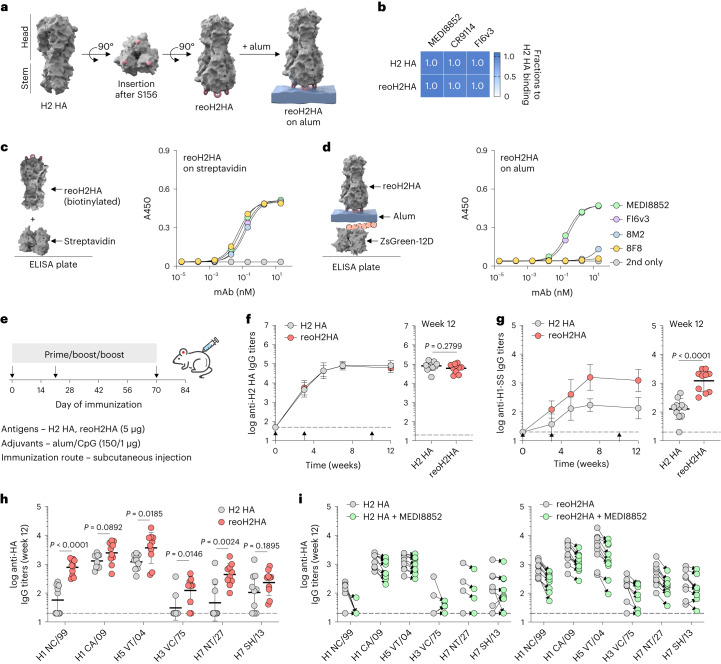
Reorientation of H2 HA enabled immunofocusing on HA-stem. **a**, Insertion of oligoD into the head of H2 HA (A/Japan/305/1957) after residue S156 (Protein Data Bank ID: 2WRE). Three oligoD motifs on HA-head allowed for its tri-valent anchoring on alum. **b**, BLI binding analysis of H2 HA or reoH2HA with HA-stem-specific mAbs. **c**,**d**, Binding of stem-directed (MEDI8852 and FI6v3) or head-directed (8F8 and 8M2) mAbs to reoH2HA on streptavidin-coated (**c**) or alum-coated (**d**) ELISA plates. Data are presented as mean ± s.d. (*n* = 4 technical replicates). **e**, A three-dose immunization study with H2 HA or reoH2HA adjuvanted with alum/CpG in BALB/c mice. **f**,**g**, Serum H2 HA-specific (**f**) or stem-specific (**g**) IgG titers over time (*n* = 10 mice per group). Stem-specific IgG titers were measured with the H1-SS protein. Endpoint (week 12) antibody titers from the two groups are plotted on the right for comparison (each circle represents a single mouse). **h**, Cross-reactive binding of group 1 (H1 NC/99, H1 CA/09 and H5 VT/04) and group 2 (H3 VC/75, H7 NT/27 and H7 SH/13) HAs by week 12 antisera. Each circle represents a single mouse (*n* = 10 mice per group). **i**, Serum binding titers to group 1 and group 2 HAs in the presence of competing mAb—MEDI8852. Each circle represents a single mouse (*n* = 10 mice per group). Arrows indicate prime and boost immunizations in **f**,**g**. Dashed lines indicate the limit of quantification in **f**–**i**. Data are presented as geometric mean ± s.d. of the log_10_‐transformed values (**f**–**i**). Comparison of two groups was performed using the two-tailed Mann–Whitney *U*-test. *P* values of 0.05 or less were considered significant. Source data

**Extended Data Fig. 7:**
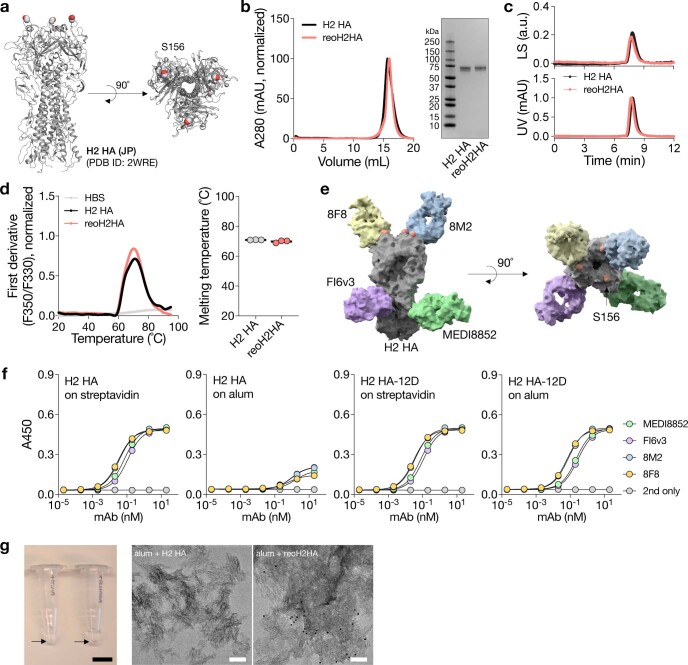
OligoD insertion into HA-head of H2 HA. a, Location of the oligoD insertion site (S156) on the head of H2 HA. b, Size-exclusion purification and gel electrophoresis of H2 HA and reoH2HA. Gels were stained with Coomassie brilliant blue. c, SEC-MALS analysis of H2 HA and reoH2HA on a Superdex 200 column. UV and LS indicate absorbance at 280 nm and light scattering signals, respectively. d, Thermal melting profiles and T_m_ of H2 HA and reoH2HA. Data are presented as mean ± s.d. in the dot plots (n = 3 samples per group). e, Epitopes targeted by head-directed (8F8 and 8M2) and stem-directed mAbs (MEDI8852 and FI6v3) on H2 HA. Red spheres indicated oligoD insertion sites (S156). f, Binding of head-directed (8F8 and 8M2) or stem-directed mAbs (MEDI8852 and FI6v3) to H2 HA or H2 HA-12D (H2 HA with 12D inserted at its C-terminus) on streptavidin-coated (left) or alum-coated (right) ELISA plates. Data are presented as mean ± s.d. (n = 4 technical replicates). g, Immunogold labeling (left, scale bar, 1 cm) and TEM imaging (right, scale bar, 100 nm) of alum complexed with H2 HA or reoH2HA. Source data

Having validated using three protein antigens that oligoD insertion enabled their binding to alum and increased their immunogenicity, we then attempted to insert oligoD into HA-head to anchor this region on alum and redirect antibody responses to the newly exposed HA-stem (Fig. 5a). We chose to insert oligoD into an H2 HA because the bulky phenylalanine (F45_HA2_) in its HA-stem expands the cross-reactivity of stem-binding B cells^36^. Although oligoD insertions into HA-head often led to substantially reduced protein expression levels (Supplementary Fig. 5a), insertion after residue S156 in the head of H2 HA (A/Japan/305/1957) was successful (Extended Data Fig. 7a) and produced reoH2HA (Extended Data Fig. 7b,c). ReoH2HA maintained the thermal stability of its wild-type counterpart (Extended Data Fig. 7d), including in the presence of alum (Supplementary Fig. 5b). The insertion into the head of H2 HA also did not appreciably affect the binding profiles (Supplementary Fig. 5c and Fig. 5b) or affinities (Supplementary Fig. 5d–f) of stem-directed mAbs against the protein. To validate the ‘upside-down’ orientation of oligoD-modified HA on alum, we measured binding of head-directed and stem-directed mAbs (Extended Data Fig. 7e) to reoH2HA on streptavidin-coated or alum-coated ELISA plates (Fig. 5c,d). When C-terminal biotinylated H2 HA or reoH2HA was adsorbed on streptavidin-coated ELISA plates (Extended Data Fig. 7f and Fig. 4c), both head-directed (8F8 and 8M2) and stem-directed (MEDI8852 and FI6v3) mAbs showed similar high-affinity binding to these antigens, suggesting that oligoD insertion did not affect the antigenicity of H2 HA. Because H2 HA adsorbed poorly on alum, we created H2 HA with 12D inserted at its C-terminus as an ‘upright’ control and found identical mAb-binding profiles between streptavidin-coated and alum-coated plates (Extended Data Fig. 7f). In contrast, only stem-directed mAbs bound to reoH2HA when it was adsorbed on alum (Fig. 5d). Loss of binding of head-directed mAbs suggests that reoH2HA adopted an ‘upside-down’ configuration on alum, where epitopes on HA-head were no longer accessible. We also validated binding of reoH2HA on alum via immunogold labeling, where we observed decoration of alum by reoH2HA but not H2 HA by transmission electron microscopy (Extended Data Fig. 7g).

**Extended Data Fig. 8:**
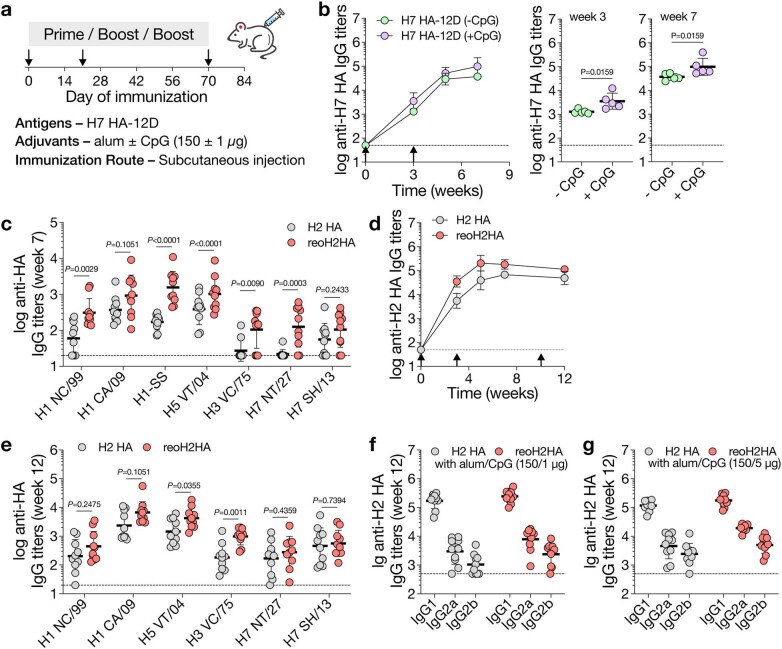
Immunization of oligoD-modified HA. a, A prime-boost immunization study with H7 HA-12D adjuvanted with alum alone or alum/CpG via subcutaneous injection in BALB/c mice (n = 5 mice per group). b, Serum H7 HA-specific IgG titers over time. Antibody titers of weeks 3 and 7 from the two groups are plotted on the right for comparison. c, Cross-reactive binding of different group 1 (H1 NC/99, H1 CA/09, H2 JP/57, H5 VT/04) and group 2 HAs (H3 VC/75, H7 NT/27, H7 SH/13) by week-7 antisera (n = 10 mice per group from the immunization study in Fig. 5e). d, Serum H2 HA-specific IgG titers over time (n = 10 mice per group from the immunization study in Fig. 6a). e, Cross-reactive binding of different group 1 (H1 NC/99, H1 CA/09, H2 JP/57, H5 VT/04) and group 2 HAs (H3 VC/75, H7 NT/27, H7 SH/13) by week-12 antisera (n = 10 mice per group from the immunization study in Fig. 6a). f,g, IgG subtypes of week-12 sera raised from low- (f) or high-dose (g) CpG coupled with alum. Dashed lines indicate the limit of quantification in b-g. IgG titers are presented as the geometric mean of the log_10_-transformed values in b-g. Comparison of two groups was performed using the two-tailed Mann–Whitney *U*-test in b,c,e. *P* values of 0.05 or less were considered significant. Source data

We next immunized mice with H2 HA or reoH2HA adjuvanted with alum and CpG oligodeoxynucleotide (Fig. 5e). We chose this combined alum/CpG adjuvant because we found in an immunization study with another HA (A/Shanghai/2/2013) that it further elevated the overall antibody titers compared to alum alone (Extended Data Fig. 8a,b). After three injections, mice from both groups developed robust H2 HA-specific IgG responses (Fig. 5f). Despite similar antigen-specific titers, reoH2HA elicited 10-fold higher antibody titers against HA-stem, as measured by binding of antisera to the H1-stabilized stem (H1-SS) protein^28^ (Fig. 5g). To test whether stem binding translated to increased cross-reactivity, we measured binding of antisera to different group 1 and group 2 HAs. After two doses, antisera from mice immunized with reoH2HA showed higher cross-reactive titers than those immunized with H2 HA (Extended Data Fig. 8c). Even after three doses, mice immunized with H2 HA mostly cross-reacted with half of the HAs that we tested (H1 CA/09, H5 VT/04 and H7 SH/13) although with relatively low titers. In contrast, almost all mice immunized with reoH2HA developed cross-reactive responses against both group 1 and group 2 HAs tested (Fig. 5h). To further evaluate if cross-reactive responses were focused, at least partially, on neutralizing epitopes of HA-stem, we analyzed whether antisera competed for binding with a known stem-specific broadly neutralizing antibody (bnAb) (MEDI8852). Compared to the H2 HA group, antisera against reoH2HA showed greater competition with MEDI8852 (Fig. 5i). Similarly, there was also stronger competition with a pan-H1, anchor-directed antibody (222-1C06) with antisera from the reoH2HA group (Supplementary Fig. 6a). As a control, antisera from neither group showed much competition for binding with another broadly protective antibody (FluA-20) that targets the trimer interface of HA (Supplementary Fig. 6b). These results indicate that alum-bound reoH2HA directed antibody responses away from the head and toward the stem region of H2 HA.

**Fig. 6:**
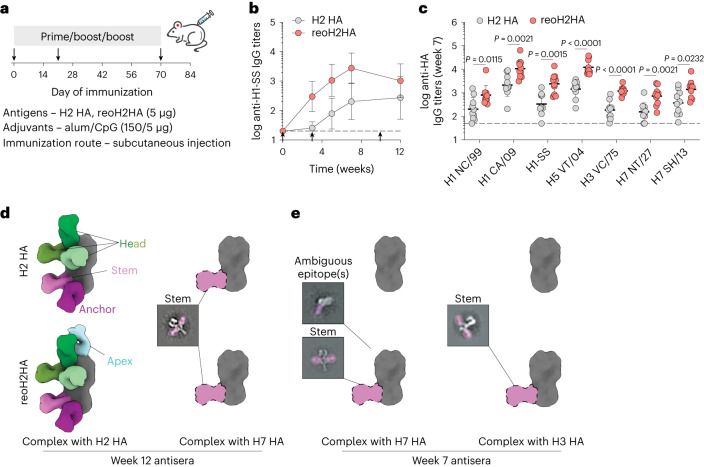
Cross-reactive antibodies elicited by reoH2HA recognize group 2 HA-stem. **a**, A three-dose immunization study with H2 HA or reoH2HA adjuvanted with a higher dose of CpG with alum in BLAB/c mice. **b**, Serum stem-specific IgG titers over time (*n* = 10 mice per group). Arrows indicate prime and boost immunizations in **b**. **c**, Cross-reactive binding of group 1 (H1 NC/99, H1 CA/09 and H5 VT/04) and group 2 (H3 VC/75, H7 NT/27 and H7 SH/13) HAs by week 7 antisera. Each circle represents a single mouse (*n* = 10 mice per group). Dashed lines indicate the limit of quantification in **b**,**c**. Data are presented as geometric mean ± s.d. of the log_10_‐transformed values (**b**,**c**). Comparison of two groups was performed using the two-tailed Mann–Whitney *U*-test. *P* values of 0.05 or less were considered significant. **d**,**e**, nsEMPEM analyses for week 12 (**d**) or week 7 (**e**) pooled antisera raised against H2 HA (top row) or reoH2HA (bottom row). Composite maps generated from negative-stain reconstructions of polyclonal immune complexes with H2 HA, H7 HA or H3 HA are shown for each group. Representative 2D class averages with the polyclonal Fab labeled are shown for immune complexes in low abundance, in which dotted lines highlight the likely position for the Fab population. Source data

To further boost cross-reactive responses, we immunized mice with H2 HA or reoH2HA adjuvanted with a higher dose of CpG (Fig. 6a). Similar to the previous immunization, both antigens induced robust H2 HA-specific IgG responses (Extended Data Fig. 8d), whereas reoH2HA elicited greater stem-directed responses (Fig. 6b) and cross-reactivity toward both group 1 and group 2 HAs (Fig. 6c and Extended Data Fig. 8e). Antisera raised against reoH2HA also showed stronger competition with MEDI8852 (Supplementary Fig. 6c) and 222-1C06 (Supplementary Fig. 6d) than those against H2 HA did, and both groups showed negligible competition with FluA-20 (Supplementary Fig. 6e). Despite an increase in the CpG dose (1 µg to 5 µg), the overall antibody responses were still Th2-biased given the use of high-dose alum (150 µg) (Extended Data Fig. 8f,g).

**Extended Data Fig. 9:**
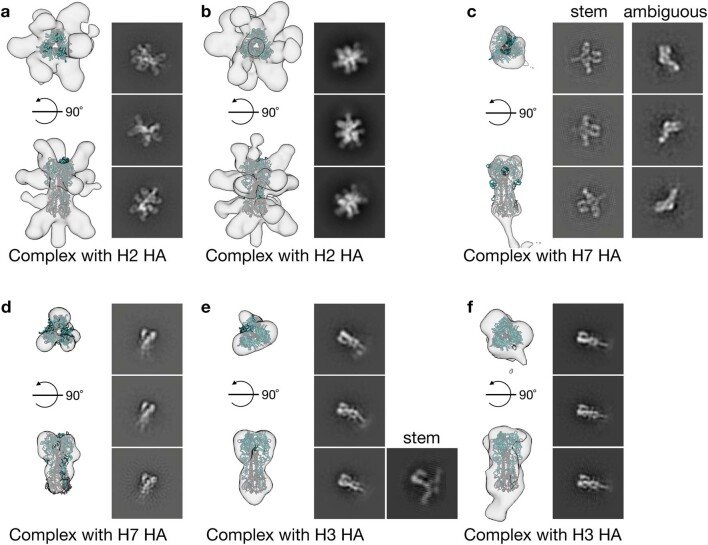
Polyclonal epitope mapping with negative-stain electron microscopy (nsEMPEM). a-f, 3D negative-stain reconstructions with representative 2D class averages of polyclonal immune complexes with H2 HA (a,b), H7 HA (c,d) or H3 HA (e,f). Immune complexes generated with antisera against H2 HA or reoH2HA were shown in a,d,f or b,c,e, respectively.

To characterize epitopes targeted by cross-reactive antisera, we created polyclonal immune complexes (Supplementary Fig. 7a,b) and performed nsEMPEM^38^ (Supplementary Fig. 7c). After three immunizations, both antigens induced antibodies binding to head, stem and anchor epitopes of H2 HA (Fig. 6d and Extended Data Fig. 9a,b), whereas reoH2HA also elicited antibodies targeting the apex of intact HA trimers (Extended Data Fig. 9b), possibly due to introduction of a neo-antigen by oligoD near this region. We also observed low levels of cross-reactive, stem-directed antibodies against a group 2 HA (H7 HA) from both groups. We then performed additional nsEMPEM analysis with two group 2 HAs using week 7 antisera, where we observed the highest stem-directed IgG titers from the reoH2HA group (Fig. 6c). When complexed with H7 HA, reoH2HA elicited antibodies targeting HA-stem and another ambiguous epitope (Extended Data Fig. 9c), whereas responses to these epitopes were not observed in antisera against H2 HA (Fig. 6e and Extended Data Fig. 9d). Notably, immunization with reoH2HA also induced low levels of cross-reactive antibodies targeting H3 HA-stem (Extended Data Fig. 9e) despite the lowest cross-reactive titers toward H3 HA, whereas immunization with H2 HA did not (Extended Data Fig. 9f). Taken together with ELISA data, reoH2HA stimulated a stem-directed antibody response with higher magnitude, breadth and affinity than H2 HA did.

**Extended Data Fig. 10:**
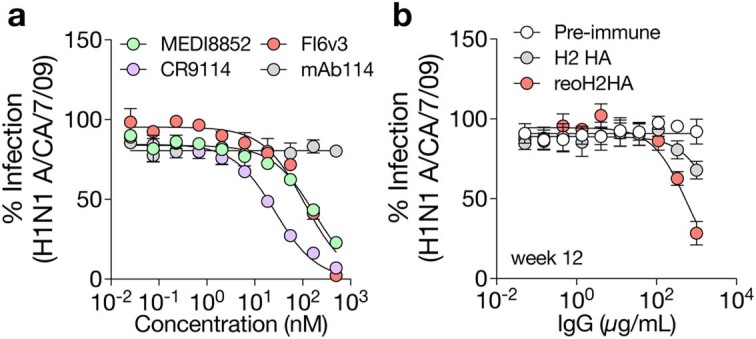
Cross-neutralization of a heterosubtypic H1N1 virus. a, Validation of the IAV microneutralization assay with three stem-directed bnAbs (MEDI8852, CR9114 and FI6v3) against authentic A/California/7/2009 (H1N1 A/CA/7/09) viruses. A GP-specific mAb (mAb114) served as a negative control. Data are presented as mean ± s.d. (n = 4 technical replicates). b, Neutralization of H1N1 A/CA/7/09 viruses by IgG purified from pooled antisera against H2 HA or reoH2HA. IgG purified from pre-immune sera served as a control. Week-12 antisera raised from Fig. 6a were used for analysis. Data are presented as mean ± s.d. (n = 4 technical replicates). Source data

Considering the generally weak neutralizing activity of stem-directed antibodies and the limited availability of sera, we pooled antisera from each group and purified IgG to assess the cross-neutralizing activity against A/California/7/09 (H1N1) viruses (Extended Data Fig. 10a), because we observed the highest cross-reactive titers against this corresponding H1 HA. Whereas IgG purified from the H2 HA group did not show any significant cross-neutralization at all concentrations, IgG purified from the reoH2HA group exhibited modest cross-neutralizing activity against this heterosubtypic H1N1 strain (Extended Data Fig. 10b).

## Discussion

It remains challenging to acquire durable and protective immunity from vaccinations against viruses with high sequence variability, such as influenza and SARS-CoV-2. However, the discovery of bnAbs that target conserved epitopes on viral glycoproteins suggests the possibility of developing universal vaccines against these targets and has prompted the development of epitope-focused vaccine candidates to direct antibody responses toward bnAb epitopes^7,39^. In the case of influenza virus, intense efforts to focus antibody responses to HA-stem include cross-strain boosting^15–18^, mosaic display^19,20^, epitope resurfacing^21^, epitope masking^23–25^ and protein dissection^26–29^. Although these diverse immunofocusing approaches have led to substantial progress toward broadening the breadth of the antibody response, it has not been possible to elicit an antibody response with a single immunogen that consistently cross-reacts with HAs from both group 1 and group 2 influenza A subtypes.

Here we introduce a new approach to immunofocusing—antigen reorientation. Our hypothesis was that the reorientation of antigens could alter epitope accessibility and redirect the immune response to less dominant but more desirable epitopes. Using influenza HA as a model, we reoriented the full-length HA ectodomain in an ‘upside-down’ configuration facilitated by its binding to alum, to redirect antibody responses away from the variable HA-head and toward the highly conserved HA-stem. We envisioned that this approach could increase the exposure of the poorly accessible HA-stem that was originally occluded by HA-head on influenza virions^30,31^ while simultaneously sterically decreasing the exposure of HA-head—in the context of an intact HA trimer that preserves the native structure of HA-stem.

To test this, we genetically inserted oligoD into HA-head and found that this modification enhanced antigen-binding to alum in an ‘upside-down’ configuration, likely through electrostatic interactions. Compared to existing immunofocusing approaches, our strategy simultaneously preserved the native structure and conformational epitopes of HA and mitigated the immunodominance of HA-head by tethering it to alum. Immunization with wild-type H2 HA on alum induced an antibody response that cross-reacted with several of the HAs tested, consistent with previous results^40^. By contrast, reoH2HA on alum elicited higher titers of cross-reactive antibodies against all group 1 and group 2 HAs tested (Fig. 6c), in support of our hypothesis that antigen reorientation redirected antibody responses to the HA-stem region. Data from the competition assay (Fig. 5i and Supplementary Fig. 6c) and nsEMPEM (Fig. 6d,e) further revealed that antibodies targeting HA-stem epitopes afforded cross-reactivity toward two group 2 HAs (H3 and H7 HAs). We also observed cross-reactive antibodies targeting an ambiguous epitope on H7 HA (Extended Data Fig. 9c), which warrants future investigations. Taken together, increased exposure and accessibility of HA-stem in the reoriented HA directed a fraction of the overall response to important stem epitopes.

Our results provide proof of concept that the reorientation of viral antigens leads to a stem-directed cross-reactive antibody response. Although antisera raised against reoH2HA only showed modest cross-neutralizing activity, they may still confer protection via Fc-mediated effector functions, as demonstrated in the case of H1-SS nanoparticles^28^. Whereas IgG2 correlates with stronger effector functions than IgG1 in mice, use of alum/CpG in our studies drove a Th2-biased response with much higher titers of IgG1 than IgG2 (Extended Data Fig. 8f,g). Therefore, it will be important to explore different ratios and formulations of adjuvants with the aim of boosting a balanced Th1/Th2 response with broad cross-reactivity. Because our reorientation approach relies on antigen binding to alum, formulation of reoH2HA with emulsion-based adjuvants remains a challenge. It remains to be determined whether cross-reactive antibody responses elicited by reoriented antigens protect against lethal challenges by direct viral neutralization or Fc-mediated mechanisms.

The simplicity of our approach makes it readily generalizable to other antigens because it only requires molecular cloning and screening of insertion sites. Meanwhile, existing methods for attaching antigens to alum require additional synthesis of linker molecules followed by chemical conjugation and subsequent purification steps^35,41^, which may face challenges in controlling mass transport and efficient mixing during large-scale manufacture. Importantly, identifying sites where insertion of oligoD is permissive for protein expression in mammalian cells will likely be required to generate antigens with preserved native structures^42^. The tri-valent anchoring afforded by three distinct oligoD motifs within each HA trimer rigidifies the antigen–alum binding interface and reduces the possibility of antigens ‘lying down’ on alum. In addition, conceptually similar motifs that harness electrostatic interactions (for example, oligomeric glutamate residues) and other protein tags or binding partners^43–45^ may substitute or could be combined with oligoD for antigen reorientation and high-resolution immunofocusing purposes.

In summary, we introduce a generalizable immunofocusing strategy that enables the control of antigen orientation on alum by oligoD insertion. Our results showed consistent cross-reactivity toward group 1 and group 2 HAs with a single immunogen (reoH2HA) that encodes HA from a single influenza subtype. This approach has the potential to accelerate the development of epitope-focused vaccines against a broad range of viruses and other infectious agents.

## Methods

### Cell lines

HEK-293T and MDCK.2 cells were purchased from the American Type Culture Collection and maintained in D10 medium—DMEM (Corning) supplemented with 10% FBS (GeminiBio) and 1% penicillin–streptomycin–L-glutamine (GeminiBio). HeLa-ACE2/TMPRSS2 cells were provided by Jesse Bloom at the Fred Hutchinson Cancer Research Center as a generous gift and were maintained in D10 media. Expi-293F cells were purchased from Thermo Fisher Scientific and maintained in Freestyle293/Expi-293 media (2:1, v/v, Thermo Fisher Scientific) in polycarbonate shaking flasks (TriForest Labware). Stellar and BL21(DE3) competent cells were purchased from Takara Bio and Thermo Fisher Scientific, respectively.

### Antibodies

mAbs against Ebola GP (mAb114, c13C6, ADI-15742, KZ52 and ADI-16061), SARS-CoV-2 spike (COVA2-15, CB6 and CR3022) or influenza hemagglutinin (CH65, H2897, 6649, MEDI8852, CR9114, FI6v3, 8F8, 8M2, 222-1C06 and FluA-20) were expressed in Expi-293F cells via transient transfection.

Mouse anti-His Tag antibody (clone J099B12, BioLegend, 652502), goat anti-mouse IgG, HRP conjugated (clone Poly4053, BioLegend, 405306), goat anti-mouse IgG1-HRP (SouthernBiotech, 1070-05), goat anti-mouse IgG2a-HRP (SouthernBiotech, 1083-05), goat anti-mouse IgG2b-HRP (SouthernBiotech, 1093-05), rabbit anti-human IgG, HRP conjugated (Abcam, ab6759) and goat anti-rabbit IgG, HRP conjugated (Invitrogen, G-21234) were used as detecting antibodies for western blot analysis or ELISAs. Goat anti-human IgG, gold-conjugated (Electron Microscopy Sciences, 25208) was used for immunogold labeling. Mouse anti-influenza A nucleoprotein antibody (clones A1, A3 blend, Sigma-Aldrich, MAB8251) and rabbit anti-influenza A nucleoprotein antibody (clone HL1078, Invitrogen, MA5-42363) were used for influenza A virus microneutralization assays. For the analysis of GC responses, we stained cells with Ghost Dye Violet 510 (Tonbo Biosciences), anti-mouse CD16/32 (clone 2.4G2, BD Biosciences, 553142), CD3 (clone 17A2, BioLegend, 100216), CD4 (clone GK1.5, BioLegend, 100469), CXCR5 (clone L138D7, BioLegend, 145529), PD1 (clone 29F.1A12, BioLegend, 135228), CD19 (clone 6D5, BioLegend, 115534), CD95 (clone Jo2, BD Biosciences, 557653), CD38 (clone 90, BD Biosciences, 740245) and IgG (clone A85-1, BD Biosciences, 550083).

### Antigen and antibody cloning

DNA encoding the Ebola GP (Mayinga, Zaire, 1976) ectodomain with the mucin-like domain deleted (residues 1–308 and 491–656) and the transmembrane domain replaced with a GCN4 (ref. ^46^) or foldon trimerization^47^ domain followed by an Avi-Tag^48^ and a hexahistidine tag was cloned into a mammalian expression vector (pADD2) by In-Fusion (Takara Bio). Similarly, DNAs encoding SARS-CoV-2 spike (residues 1–1,143 from the Wuhan-Hu-1 genome sequence, GenBank, MN9089473) or influenza hemagglutinins (HAs of H1N1 A/New Caledonia/20/99, H1N1 A/California/07/2009, H2N2 A/Japan/305/1957, H5N1 A/Vietnam/1203/2004, H3N2 A/Victoria/3/1975, H7N7 A/FPV/Dutch/1927 and H7N9 A/Shanghai/2/2013) were cloned into the pADD2 vector with a GCN4 or foldon trimerization domain followed by an Avi-Tag and a hexahistidine tag on the C-terminus. DNA encoding Gen6′ H1-SS was cloned into the pADD2 vector with a GCN4 trimerization domain followed by an Avi-Tag and a hexahistidine tag on the C-terminus. OligoD was inserted at the C-terminus after the hexahistidine tag in pADD2 expression plasmids. OligoD was also inserted into the flexible loop regions on Ebola GP or head regions of H1 HA (A/New Caledonia/20/99 or A/California/07/2009), H2 HA (A/Japan/305/1957), H5 HA (A/Vietnam/1203/2004), H3 HA (A/Victoria/3/1975) or H7 HA (A/FPV/Dutch/1927 or A/Shanghai/2/2013) for screening purposes. DNA encoding ACE2–Fc fusion or ZsGreen with an Avi-tag, a hexahistidine tag and oligoD insertion to the C-terminus (ZsGreen-Avi-His-12D) was cloned into the pADD2 vector. Superfolder green fluorescent protein with a GCN4, an Avi-tag and a hexahistidine tag on the C-terminus (GFP-GCN4-Avi-His) was cloned into a pET28a bacterial expression vector^49^. DNA fragments encoding the variable heavy chain (HC) and light chain (LC) of antibodies were inserted into an expression plasmid encoding VRC01 HC and LC constant domains by In-Fusion. All plasmid sequences were confirmed by Sanger sequencing (Sequetech). For transfection, plasmids were transformed into Stellar cells, isolated by Maxiprep kits (NucleoBond Xtra Maxi kit, Macherey Nagel), filtered through a sterile 0.45-µm membrane in a biosafety cabinet and stored at −20 °C.

### Protein expression and purification

All antigens and antibodies were expressed in Expi-293F cells. Expi-293F cells were cultured at 37 °C under constant shaking (120 r.p.m.) in a humidified CO_2_ (8%) incubator. Expi-293F cells were transfected at a density of 3–4 × 10^6^ cells per milliliter. For 200-ml transfection of antigen proteins, the transfection mixture was made by adding 120 µg of plasmid DNA (from Maxiprep) to 20 ml of expression media, followed by dropwise addition of 260 µl of FectoPro transfection reagent (Polyplus) with vigorous mixing. For antibody production in 200 ml of Expi-293F cells, the transfection mixture contained 60 µg of LC plasmid DNA and 60 µg of HC plasmid DNA. Transfection mixtures were incubated at room temperature for 10 min before being transferred to Expi-293F cells. D-glucose (4 g L^−1^, Sigma-Aldrich) and valproic acid (3 mM, Acros Organics) were added to the cells immediately after transfection to increase recombinant protein production. Cells were boosted again with D-glucose 3 d after transfection and harvested on day 4 by centrifugation at 7,000*g* for 5 min. The supernatant was filtered through a 0.22-µm membrane for subsequent purification. Biotinylated proteins were expressed using the same protocol but in the presence of the BirA enzyme. For screening purposes, we expressed proteins in small scales (5–10 ml of Expi-293F cells) and analyzed the supernatant with western blots. We used mAb114 (0.5 µg ml^−1^) or anti-His Tag antibody (1:4,000) to detect Ebola GP or influenza HAs, respectively.

Antigen proteins with hexahistidine tags were purified with HisPur Ni-NTA resin (Thermo Fisher Scientific). In brief, the filtered supernatant from Expi-293F cells was mixed with Ni-NTA resin (1 ml of resin per liter of supernatant) and incubated at 4 °C overnight. The mixture was then passed through a gravity-flow column, washed with 20 mM imidazole in HEPES buffer saline (HBS) (20 mM HEPES, pH 7.4, 150 mM NaCl) and then eluted with 250 mM imidazole in HBS. Elution was concentrated with centrifugal filters (30 kDa or 50 kDa molecular weight cut-off (MWCO), MilliporeSigma) and buffer exchanged to HBS for size-exclusion chromatography using a Superose 6 (Increase 10/300 GL, Cytiva) or Superdex 200 column (Increase 10/300 GL, Cytiva) on an ÄKTA Protein Purification System (Cytiva). Peak fractions were pooled, concentrated, buffer exchanged to HBS with 10% glycerol and filtered through a 0.22-µm membrane.

All antibodies and ACE2–Fc were purified with MabSelect PrismA protein A chromatography. Filtered supernatant from Expi-293F cells was directly applied to a MabSelect PrismA column (Cytiva) on an ÄKTA Protein Purification System. The column was washed with HBS, and then antibodies were eluted with glycine (100 mM, pH 2.8) to HEPES buffer (1 M, pH 7.4). Fractions were concentrated and buffer exchanged to HBS with 10% glycerol. Fragment antigen binding (Fab) was prepared by digesting antibodies (IgG) with Lysyl endopeptidase (Wako Chemicals). After digestion, Fabs were purified with Protein-A Agarose (Thermo Fisher Scientific) and buffer exchanged to HBS with 10% glycerol.

BL21(DE3) competent cells were transformed with the pET28a plasmid encoding GFP-GCN4-Avi-His and induced at an optical density of 0.6 with isopropyl *β*-d-*1*-thiogalactopyranoside (IPTG, 1 mM) for 3 h at 37 °C. Cells were harvested by centrifugation at 8,000*g* for 5 min, resuspended in HBS with protease inhibitors (Halt Protease and Phosphatase Inhibitor Cocktail, Thermo Fisher Scientific) and lysed by sonication. Cell lysates were centrifuged at 16,000*g* for 60 min, and the supernatant was further purified with Ni-NTA resin, as described above.

The concentration of all proteins was determined by absorbance at 280 nm (A280), and the purity was assessed by protein gel electrophoresis. Protein samples were flash frozen in liquid nitrogen and stored at −20 °C.

### Size-exclusion chromatography–multi-angle light scattering analysis

Size-exclusion chromatography–multi-angle light scattering (SEC-MALS) analysis of wild-type and oligoD-modified proteins was performed on a 1260 Infinity II high-performance liquid chromatography system (Agilent) coupled with a miniDAWN and Optilab detectors (Wyatt Technologies) for light scattering and refractive index analysis. Purified protein samples (0.2–0.5 mg ml^−1^) were loaded onto a Superdex 200 column (Increase 3.2/300, Cytiva) sequentially for analysis. ASTRA software (version 7.3.2.21, Wyatt Technologies) was used for quantitative analysis of the molar mass of antigen proteins (Supplementary Table 1).

### Differential scanning fluorimetry

Thermal melting profiles of proteins were measured by differential scanning fluorimetry on a Prometheus NT.48 instrument (NanoTemper). Protein samples (0.1 mg ml^−1^) were loaded into glass capillaries (NanoTemper) and then subjected to a temperature gradient from 20 °C to 95 °C at a heating rate of 1 °C per minute. Alternatively, protein samples (0.1 mg ml^−1^) were pre-mixed with alum (10 mg ml^−1^, Alhydrogel, InvivoGen) at a ratio of 1:10 (protein:alum, w/w) for 30 min at room temperature before loading into glass capillaries. HBS and alum (diluted to 1 mg ml^−1^ in HBS) were also loaded into glass capillaries and measured as controls. Intrinsic fluorescence (350 nm and 330 nm) was recorded as a function of temperature. Thermal melting curves were plotted using the first derivative of the ratio (350 nm/330 nm). Melting temperatures were calculated automatically by the instrument (PR.ThermControl software, version 2.3.1) and represented peaks in the thermal melting curves.

### BLI

BLI experiments were performed on an Octet RED96 system (FortéBio). All samples were diluted with the Octet buffer (DPBS with 0.05% Tween 20 and 0.1% BSA), and assays were performed under agitation (1,000 r.p.m.). mAbs (for example, mAb114, c13C6, ADI-15742, KZ52 and ADI-16061 for Ebola GP; COVA2-15, CB6 and CR3022 for spike; CH65, H2897, 6649, MEDI8852, CR9114 and FI6v3 for H1 HA; all at 200 nM) or ACE2–Fc (200 nM) were loaded onto anti-human IgG Fc capture (AHC) biosensors (FortéBio) and then dipped into antigen solutions (for example, GP, spike and H1 HA at 150 nM or 200 nM) for binding analysis, followed by dissociation into the Octet buffer. Alternatively, biotinylated antigens (for example, H2 HA or reoH2HA at 200 nM) were loaded onto streptavidin biosensors (Sartorius) and then dipped into antibody solutions (for example, MEDI8852, CR9114 and FI6v3 at 200 nM) for binding analysis, followed by dissociation into the Octet buffer. Serial dilutions of Fabs (for example, MEDI8852, CR9114 and FI6v3 starting at 200 nM) were used to measure the affinity (K_D_) for biotinylated antigens. Data were processed by Data Analysis software (version 9.0.0.15, FortéBio) and then plotted. Shifts in nanometers of mAb binding to wild-type proteins were used as the maximal binding value (1.0), and shifts from oligoD-modified proteins were normalized as a fraction of the maximal binding value.

### Antigen–alum binding assays

Wild-type GP or oligoD-modified GP was first incubated with alum (protein:alum, 1:10, w/w) for 30 min in PBS at room temperature, and then naive mouse serum was added to the mixture to a final concentration of 10% (v/v). The mixture with alum alone or wild-type GP alone served as controls. Mixtures were further incubated at 37 °C under constant shaking (220 r.p.m.) on an orbital shaker for 24 h before being centrifuged to pellet alum (10,000*g* for 5 min). The supernatant was collected for ELISA to measure the concentration of unbound protein antigens. Alum pellets were rinsed extensively with PBS and re-pelleted again (twice) by centrifugation and then resuspended in SDS-PAGE sample loading buffer for western blot analysis to determine the amount of alum-bound protein.

To measure the amount of unbound GP, we coated Nunc 96-well MaxiSorp plates (Thermo Fisher Scientific) with mAb114 (2 µg ml^−1^ in DPBS, 50 µl per well) for 1 h at room temperature. Plates were washed three times with Milli-Q water (300 µl) using a plate washer (ELx405, BioTek) and then blocked with ChonBlock (100 µl per well, Chondrex) overnight at 4 °C. For subsequent steps, all dilutions were made in DPBS with 0.05% Tween 20 and 0.1% BSA, and ELISA plates were rinsed with PBST (PBS with 0.1% Tween 20, 300 µl, three times) in between steps. Antigens with proper dilutions were added to the plate and detected by the mouse anti-His tag antibody (1:4,000). Goat anti-mouse IgG, HRP-conjugated (1:4,000), was then added for 1-h incubation before rinsing with PBST six times. ELISA plates were developed with the 3,3′,5,5′-tetramethylbenzidine (TMB) substrate (1-Step Turbo-TMB, Thermo Fisher Scientific) for 6 min and terminated with sulfuric acid (2 M). Absorbance at 450 nm was recorded on a microplate reader (Synergy HT, BioTek). GP with a series of known concentrations was used to establish a standard curve, and the amount of antigen was quantified by fitting the absorbance values to this curve.

For western blot analysis, alum pellets were resuspended in sample loading buffer and boiled at 95 °C for 10 min before being applied to a pre-cast polyacrylamide gel (4–20% Mini-PROTEAN, Bio-Rad). Proteins were then transferred to a nitrocellulose membrane using the Trans-Blot Turbo transfer system (Bio-Rad). Blots were blocked in PBST with 10% non-fat dry milk (Bio-Rad), incubated with mAb114 (0.5 µg ml^−1^ in PBST with 10% non-fat dry milk) and then detected with rabbit anti-human IgG, HRP-conjugated (1:4,000 in PBST with 10% non-fat dry milk). Luminescent signals were developed using a luminol-based substrate (Pierce ECL, Thermo Fisher Scientific) for imaging on a chemiluminescence imager (GE Amersham Imager 600).

### Immunogold staining

Similar to the antigen–alum binding assay, GP, GP-12D, H2 HA or reoH2HA was incubated with alum (protein:alum, 1:10, w/w) in the presence of naive mouse serum (10%, v/v, final concentration) for 24 h. The mixture with alum alone and alum plus naive mouse serum served as controls. After incubation, alum was separated by centrifugation (10,000*g* for 5min), followed by extensive rinsing with DPBS. After repeating this step three times, alum pellets were resuspended in the blocking buffer (PBST with 1% BSA) for incubation overnight. The next day, antigen–alum complexes were stained with mAb114 or MEDI8852 (20 nM, diluted in PBST with 1% BSA) for GP or HA proteins, respectively, for 10 min. After rinsing three times with DPBS, samples were further stained with goat anti-human IgG, gold-conjugated (1:10 in PBST with 1% BSA), for 10 min, followed by rinsing with DPBS six times. Samples were then pipetted onto a carbon-coated copper grid (Ted Pella) and incubated for 5 min. Grids were rinsed with DPBS six times and dried overnight. Images were acquired on a Morgagni microscope (FEI) operating with an acceleration voltage of 100 kV.

### Streptavidin-coated and alum-coated ELISA

To investigate antigen orientation on alum, we measured mAb binding in streptavidin-coated or alum-coated ELISAs. For streptavidin-coated ELISAs, Nunc 96-well MaxiSorp plates were coated with streptavidin (4 µg ml^−1^ in DPBS, 60 µl per well) for 1 h at room temperature. These plates were washed three times with Milli-Q water and then blocked with ChonBlock (120 µl per well) overnight at 4 °C. For subsequent steps, all dilutions were made in DPBS with 0.05% Tween 20 and 0.1% BSA, and ELISA plates were rinsed with PBST in between steps. Biotinylated H2 HA-12D or reoH2HA (2 µg ml^−1^) were added to the plates and incubated for 1 h at room temperature. mAbs (8F8, 8M2, MEDI8852 and FI6v3) were serially diluted (10-fold dilution starting from 20 nM) and then added to the ELISA plates for 1-h incubation at room temperature. Rabbit anti-human IgG, HRP-conjugated (1:4,000), was added for 1-h incubation before rinsing with PBST six times.

For alum-coated ELISA, Nunc 96-well MaxiSorp plates were coated with ZsGreen-Avi-His-12D (abbreviated as ZsGreen-12D, 4 µg ml^−1^ in DPBS, 60 µl per well) for 1 h at room temperature. These plates were washed three times with Milli-Q water and then blocked with ChonBlock overnight at 4 °C. For subsequent steps, all dilutions were made in DPBS with 0.05% Tween 20 and 0.1% BSA unless otherwise noted, and ELISA plates were rinsed with PBST in between steps. Alum (100 µg ml^−1^ in HBS) was added to the plates and incubated for 1 h at room temperature. After rinsing, H2 HA-12D or reoH2HA (2 µg ml^−1^) was added to the plates and incubated for 1 h at room temperature. mAbs were serially diluted (10-fold dilution starting from 20 nM) and then added to the ELISA plates for 1-h incubation at room temperature. Rabbit anti-human IgG, HRP-conjugated (1:4,000), was added for 1-h incubation before rinsing with PBST six times.

ELISA plates were developed with the TMB substrate for 5 min and terminated with sulfuric acid. Absorbance at 450 nm was recorded on a microplate reader.

### Mouse immunization studies

All animals were maintained in accordance with the Public Health Service Policy for Humane Care and Use of Laboratory Animals under a protocol approved by the Stanford University Administrative Panel on Laboratory Animal Care (APLAC-33709). Female BALB/c mice (6–8 weeks) were purchased from The Jackson Laboratory, maintained with 12-h light/dark cycles at 65–75 °F with 40–60% humidity and randomly grouped for the studies. Antigens were mixed with adjuvants before injections, with the total volume adjusted to 100 µl with HBS. Injection mixtures were incubated at room temperature for 30 min before use.

To compare GP-12D with wild-type GP, we performed a single-dose immunization in two groups of BALB/c mice (*n* = 6) with 5 µg of protein antigens adjuvanted with 150 µg of alum via subcutaneous injection. To compare spike-12D with wild-type spike, we immunized two groups of BALB/c mice (*n* = 10) with 5 µg of protein antigens adjuvanted with 150 µg of alum via subcutaneous injection on days 0, 21 and 49. To compare H1 HA-12D with wild-type H1 HA, we immunized two groups of BALB/c mice (*n* = 10) with 5 µg of protein antigens adjuvanted with 150 µg of alum via subcutaneous injection on days 0 and 56. To examine the effect of CpG oligodeoxynucleotide, we immunized two groups of BALB/c mice (*n* = 5) with 5 µg of H7 HA-12D adjuvanted with 150 µg of alum alone or 150 µg of alum and 1 µg of CpG (ODN 1826, InvivoGen) via subcutaneous injection on days 0 and 21. To compare reoH2HA with wild-type H2 HA, we immunized two groups of BALB/c mice (*n* = 10) with 5 µg of protein antigens adjuvanted with 150 µg of alum and 1 µg of CpG via subcutaneous injection on days 0, 21 and 70. We also repeated this immunization following the same regimen but with a higher dose of CpG (5 µg) coupled with alum (150 µg). Pre-immune, interim and final blood samples were collected by retro-orbital bleeding into serum gel tubes (Sarstedt). Serum gel tubes were centrifuged at 10,000*g* for 6 min, and sera were collected and stored at −80 °C.

### Serum ELISAs

We used proteins with a different trimerization domain from the immunogens for ELISA binding analysis of antisera. Nunc 96-well MaxiSorp plates were coated with streptavidin for 1 h at room temperature. These plates were washed three times with Milli-Q water using a plate washer and then blocked with ChonBlock overnight at 4 °C. For subsequent steps, all dilutions were made in DPBS with 0.05% Tween 20 and 0.1% BSA, and ELISA plates were rinsed with PBST in between steps. Biotinylated antigens (2 µg ml^−1^) were added to the plates and incubated for 1 h at room temperature. Mouse antisera were serially diluted (five-fold dilution) and then added to the ELISA plates for 1-h incubation at room temperature. Goat anti-mouse IgG, HRP-conjugated (1:4,000), was added for 1-h incubation before rinsing with PBST six times. ELISA plates were developed with the TMB substrate for 6 min and terminated with sulfuric acid. Absorbance at 450 nm was recorded on a microplate reader.

To test the cross-reactivity toward different HAs, streptavidin-coated ELISA plates were incubated with biotinylated HAs (2 µg ml^−1^) from the following strains: A/New Caledonia/20/1999 (H1 NC/99), A/California/07/2009 (H1 CA/09), A/Japan/305/1957 (H2 JP/57), A/Vietnam/1203/2004 (H5 VT/04), A/Victoria/3/1975 (H3 VC/75), A/FPV/Dutch/1927 (H7 NT/27) and A/Shanghai/2/2013 (H7 SH/13). To deplete serum antibodies that target C-terminal tags (for example, Avi-His tags), we added GP-GCN4-Avi-His protein (2 µg ml^−1^, final concentration) to the dilution buffer when testing cross-reactivity against different HAs.

For competition ELISA assays, competing antibodies (for example, MEDI8852, 222-1C06 or FluA-20, 100 nM) were pre-incubated on the plate with antigens for 1 h before the addition of serially diluted mouse antisera.

### Pseudotyped lentiviruses and neutralization assays

EBOVs encoding a luciferase-ZsGreen reporter were produced in HEK293T cells by co-transfection of five plasmids^50^. This five-plasmid system includes a packaging vector (pHAGE-Luc2-IRES-ZsGreen), a plasmid encoding full-length Ebola GP (pCDNA3.1 EBOV FL-GP) and three helper plasmids (pHDM-Hgpm2, pHDM-Tat1b and pRC-CMV_Rev1b). One day before transfection, HEK293T cells were seeded in 10-cm TC-treated culture dishes (5 × 10^6^ cells per culture dish, Corning). Transfection mixture was prepared by adding five plasmids (10 µg of packaging vector, 3.4 µg of GP-encoding plasmid and 2.2 µg of each helper plasmid) to 1 ml of D10 medium, followed by the dropwise addition of BioT transfection reagent (30 µl, Bioland Scientific) with vigorous mixing. After 10-min incubation at room temperature, the transfection mixture was transferred to HEK293T cells in the culture dish. Culture medium was replenished 24 h after transfection, and, after another 48 h, viruses were harvested and filtered through a 0.45-µm membrane. Pseudotyped lentiviruses were aliquoted, flash frozen in liquid nitrogen, stored at −80 °C and titrated before further use.

SARS-CoV-2 spike-pseudotyped lentiviruses encoding a luciferase-ZsGreen reporter were produced using the same method but with plasmids encoding SARS-CoV-2 spike (HDM-SARS2-spike-delta21, Addgene, 155130) or its variants of concern. Wild-type pseudovirus included a D614G mutation. B.1.351 (Beta) pseudovirus included D80A, D215G, Δ240-242, K417N, E484K, N501Y, D614G and A701V mutations. B.1.617.2 (Delta) pseudovirus included T19R, T95I, G142D, Δ156–157, R158G, L452R, T478K, D614G, P681R and D950N mutations. B.1.617.2/AY1 (Delta plus) pseudovirus included V70F, A222V, W258L and K417N mutations in addition to the ones in B.1.617.2. B.1.529.1 (Omicron BA.1) pseudovirus included A67V, Δ69–70, T95I, G142D, Δ143–145, Δ211, L212I, G339D, S371L, S373P, S375F, K417N, N440K, G446S, S477N, T478K, E484A, Q493R, G496S, Q498R, N501Y, Y505H, T547K, D614G, H655Y, N679K, P681H, N764K, D796Y, N856K, Q954H, N969K and L981F mutations. B.1.529.2 (Omicron BA.2) pseudovirus included T19I, Δ24–26, A27S, G142D, V213G, G339D, S371F, S373P, S375F, T376A, D405N, R408S, K417N, N440K, S477N, T478K, E484A, Q493R, Q498R, N501Y, Y505H, D614G, H655Y, N679K, P681H, N764K, D796Y, Q954H and N969K mutations.

### Neutralization assays against pseudoviruses with mAbs

Neutralization of EBOV was validated with five Ebola GP-specific mAbs. HEK293T cells were seeded in white-walled, clear-bottom 96-well plates (Thermo Fisher Scientific or Greiner Bio-One) at a density of 20,000 cells per well 1 d before the assay (day 0). On day 1, GP-specific mAbs (2 µM in HBS with 10% glycerol) were filtered with 0.22-μm sterile membranes and diluted with D10 media to 20 nM as the starting concentration. Subsequently, mAbs were serially diluted (10-fold dilution) in D10 media and mixed with EBOV (diluted in D10 medium, supplemented with polybrene, 1:1,000, v/v) for 1 h before being transferred to HEK293T cells. On day 4, medium was removed, and 100 µl of luciferase substrates (Britelite plus, PerkinElmer) was added to each well. Luminescent signals were recorded on a microplate reader (BioTek SynergyHT or Tecan M200). Percent infection was normalized to cells only (0% infection) and virus only (100% infection) on each plate. Neutralization assays were performed in four technical replicates.

Similarly, neutralization of SARS-CoV-2 pseudoviruses was validated with ACE2–Fc or three spike-specific mAbs. HeLa-ACE2/TMPRSS2 cells were seeded at a density of 6,000 cells per well 1 d before the assay (day 0). On day 1, ACE2–Fc or spike-specific mAbs (2 µM in HBS with 10% glycerol) were filtered and diluted to 20 nM as the starting concentration. Subsequently, ACE2–Fc or mAbs were serially diluted (10-fold dilution) in D10 media and mixed with SARS-CoV-2 pseudoviruses for 1 h before being transferred to HeLa-ACE2/TMPRSS2 cells. On day 3, medium was removed, and luciferase substrates were added to each well. Luminescent signals were recorded on a microplate reader and converted to percent infection for subsequent analysis. Neutralization assays were performed in four technical replicates.

### Serum neutralization assays against pseudoviruses

Antisera were heat inactivated (56 °C, 30–60 min) before neutralization assays. Neutralization against EBOV was analyzed in HEK293T cells with mouse antisera against Ebola GP. Neutralization against SARS-CoV-2 pseudoviruses was analyzed in HeLa-ACE2/TMPRSS2 cells with mouse antisera against spike. In brief, cells were seeded in white-walled, clear-bottom, 96-well plates 1 d before the assay (day 0). On day 1, antisera were serially diluted in D10 media and mixed with pseudoviruses for 1 h before being transferred to cells. Assays against EBOV or SARS-CoV-2 pseudoviruses were read out with luciferase substrates 3 d or 2 d after infection, respectively. Percent infection was normalized on each plate. NT_50_ were calculated as the serum dilution, where a 50% inhibition of infection was observed. Neutralization assays were performed in technical duplicates.

### Influenza A virus microneutralization assays

A/New Caledonia/20/1999 (H1N1 A/NC/20/99) virus was a generous gift from Florian Krammer’s laboratory at the Icahn School of Medicine at Mount Sinai. A/California/07/2009 (H1N1 A/CA/7/09) virus was purchased from Microbiologics. Viral infectivity was titrated in MDCK.2 cells, and 100× 50% tissue culture infectious dose (TCID_50_) was used for influenza A virus microneutralization assays^51^.

We first validated the assay with HA-specific mAbs. MDCK.2 cells were seeded in white-walled, clear-bottom, 96-well plates at a density of 20,000 cells per well 1 d before the assay (day 0). On day 1, mAbs were filtered and serially diluted in infection assay media (IAM) (Eagle’s minimum essential medium supplemented with 1% penicillin–streptomycin, 0.3% BSA, 0.01% FBS, 25 mM HEPES, pH 8.0, and 100 µg ml^−1^ calcium chloride). Viruses were diluted in IAM with trypsin (TPCK-treated, final concentration, 1 µg ml^−1^, Thermo Fisher Scientific) and mixed with antibody dilutions for 1 h at 37 °C before being transferred to MDCK.2 cells. After 1-h infection, we removed supernatant from all wells and rinsed cells with DPBS twice before transferring antibody dilutions for further incubation. After 48 h, we removed medium from all wells, rinsed cells with DPBS twice and fixed cells with paraformaldehyde (BioLegend). After fixation, we rinsed cells with DPBS twice and permeabilized cells with Triton X-100 (Cell Signaling Technology). For subsequent steps, detecting antibodies were diluted in DPBS with 0.05% Tween 20 and 0.1% BSA, and plates were rinsed three times with PBST in between steps. Cells were stained with mouse anti-influenza A nucleoprotein antibody (1:4,000) for 1 h, followed by goat anti-mouse IgG, HRP conjugated (1:4,000, 1 h). Plates were rinsed with PBST six times and then developed with the TMB substrate for 6 min and terminated with sulfuric acid. Absorbance at 450 nm was recorded on a microplate reader. Percent infection was normalized to cells only (0% infection) and virus only (100% infection) on each plate. Neutralization assays were performed in technical duplicates.

Antisera were first treated with receptor-destroying enzymes (Denka Seiken) at 37 °C for 20 h before adding sodium citrate to stop the treatment (2.5% w/v). Then, antisera were heat inactivated at 56 °C for 60 min and passed through centrifugal filters (Corning, 0.22 µm). Similar to mAbs, serially diluted antisera were incubated with viruses for 1 h before being transferred to MDCK.2 cells for 1-h incubation. Cells were rinsed twice and further incubated with serially diluted antisera for 48 h. For readout, cells were stained with rabbit anti-influenza A nucleoprotein antibody (1:4,000), followed by goat anti-rabbit IgG, HRP conjugated (1:4,000).

IgG was purified from pooled antisera raised against H2 HA or reoH2HA for influenza A virus microneutralization assays against H1N1 A/CA/7/09. Pooled antisera from each group were incubated with CaptureSelect IgG-Fc (multi-species) Affinity Matrix (Thermo Fisher Scientific) at 4 °C for 24 h on a rotator. Resins were transferred to centrifugal filters (0.22 µm) and centrifuged at 1,000*g* for 2 min, followed by rinsing with DPBS three times and removal of the flow-through. Mouse IgG was eluted with glycine buffer (100 mM, pH 2.8) into HEPES buffer (1 M, pH 7.4), buffer exchanged to HBS and concentrated to 2 mg ml^−1^ for influenza A virus microneutralization assays.

### nsEMPEM

Polyclonal IgG was purified from pooled mouse antisera using a Protein G column (Cytiva) before ficin digestion for 4 h at 37 °C (Pierce Mouse IgG1 Fab Preparation Kit, Thermo Fisher Scientific). Free Fab was separated from Fc using a Protein A spin column (Cytiva). Subsequently, HA trimers (5–10 µg) were complexed with polyclonal Fab at a 1:50 ratio (w/w) overnight at room temperature before purification with size-exclusion chromatography using a Superose 6 column (Increase 10/300 GL, Cytiva) against Tris-buffered saline (TBS) (20 mM Tris, pH 8.0, and 150 ml of NaCl). Fractions containing HA:Fab immune complexes were pooled and deposited for 30 s at approximately 10–15 µg ml^−1^ on freshly glow-discharged, carbon-coated copper grids (300 mesh, Electron Microscopy Sciences). Grids were then washed three times with Milli-Q water or TBS, followed by staining with uranyl formate (2%, w/v). Images were acquired at ×17,500 magnification (2.35 Å per pixel) on a Glacios transmission electron microscope (Thermo Fisher Scientific) operating at 200 kV, equipped with a Gatan K3 camera. Electron micrographs were collected in a semi-automated fashion using SerialEM before processing in RELION^52^ and cryoSPARC^53^ as previously described^38,54^.

Composite figures were created by docking 20-Å low-pass-filtered Fab volumes into 3D density corresponding to bound Fab for each complex, whereas HA is represented as a 20-Å low-pass-filtered volume. Where evidence for a Fab was only present in 2D class averages, the presumed position (based on similar 2D class averages from known HA:Fab complexes) in composite figures was marked by dotted lines around the Fab. UCSF ChimeraX was used to calculate predicted Fab footprints and generate composite figures^55^.

### Flow cytometry analysis of GC responses

To study the GC responses, we immunized BALB/c mice (*n* = 10 mice per group) with 5 µg of protein antigens (GP or GP-12D) adjuvanted with 150 µg of alum via subcutaneous injection on days 0, 7 and 14 (two groups per timepoint). On day 21, all six groups of mice and another group of naive mice were euthanized for tissue collection. Draining lymph nodes were collected and triturated through a 70-μm cell strainer to make single-cell suspensions. Cells were then stained for viability with Ghost Dye Violet 510 for 5 min on ice in PBS with EDTA (2 mM). After rinsing, cells were blocked with Fc receptor antibody (anti-CD16/32) for 5 min on ice before staining with fluorescently conjugated antibodies in FACS staining buffer (PBS, 3% FBS, 1 mM EDTA): CD3 (1:50), CD4 (1:200), CXCR5 (1:50), PD1 (1:200), CD19 (1:200), CD95 (1:200) and CD38 (1:200). Surface staining was carried out for 30 min on ice and 15 min at 37 °C, followed by fixation for 10 min at room temperature. Data were acquired on a BD LSR II flow cytometer using BD FACSDiva software (version 8.0.1) and analyzed with FlowJo software (version 10.4.0).

### Statistics and reproducibility

Western blot analysis, immunogold labeling and serological analyses were repeated at least twice with similar results, and one set of representative data is presented. Statistics were analyzed using GraphPad Prism software (version 9.3.1). Non-transformed data are presented as mean ± s.d. ELISA titers and NT_50_ were log_10_ transformed and presented as geometric mean ± s.d. Comparisons of two groups were performed using the two-tailed Mann–Whitney *U*-test. Comparisons of more than two groups were performed using one-way ANOVA followed by a Bonferroni test. Comparisons of IgG titers or NT_50_ over time were performed using two-way ANOVA followed by a Bonferroni test. *P* values of 0.05 or less are considered significant.

### Reporting summary

Further information on research design is available in the Nature Portfolio Reporting Summary linked to this article.

## Online content

Any methods, additional references, Nature Portfolio reporting summaries, source data, extended data, supplementary information, acknowledgements, peer review information; details of author contributions and competing interests; and statements of data and code availability are available at 10.1038/s41589-023-01529-6.

### Supplementary information

Supplementary Information Supplementary Figs. 1–7, Table 1 and Notes—sequences of antigens

Reporting Summary

Supplementary Data Source data for Supplementary Figs. 1–7

### Source data

Source Data Fig. 1 Statistical source data

Source Data Fig. 2 Statistical source data

Source Data Fig. 3 Statistical source data

Source Data Fig. 4 Statistical source data

Source Data Fig. 5 Statistical source data

Source Data Fig. 6 Statistical source data

Source Data Extended Data Fig. 1 Statistical source data

Source Data Extended Data Fig. 2 Statistical source data

Source Data Extended Data Fig. 3 Statistical source data

Source Data Extended Data Fig. 4 Statistical source data

Source Data Extended Data Fig. 5 Statistical source data

Source Data Extended Data Fig. 6 Statistical source data

Source Data Extended Data Fig. 7 Statistical source data

Source Data Extended Data Fig. 8 Statistical source data

Source Data Extended Data Fig. 10 Statistical source data

Source Data Fig. 1 and Extended Data Figs. 1, 2, 4, 6 and 7 Uncropped scans of blots and gels

## Data Availability

All data supporting the results of this study are available within the main text and its Supplementary Information. 3D negative-stain reconstructions for polyclonal Fab:HA complexes have been deposited in the Electron Microscopy Databank (http://www.emdataresource.org/) and are available under the following accession codes: EMD-42044, EMD-42046, EMD-42048, EMD-42056, EMD-42058 and EMD-42059. Source data are provided with this paper.
