# Supplementary material for: Vaccine design via antigen reorientation
Source: Nat Chem Biol. 2024 Jan 15;20(8):1012–21. doi: 10.1038/s41589-023-01529-6 (PMC11247139; doi:10.1038/s41589-023-01529-6)
Supplement: Supplementary file 1 — Supplementary Figs. 1–7, Table 1 and Notes—sequences of antigens [file 41589_2023_1529_MOESM1_ESM.pdf]

nature chemical biology

Article

https://doi.org/10.1038/s41589-023-01529-6

Vaccine design via antigen reorientation

In the format provided by the authors and unedited

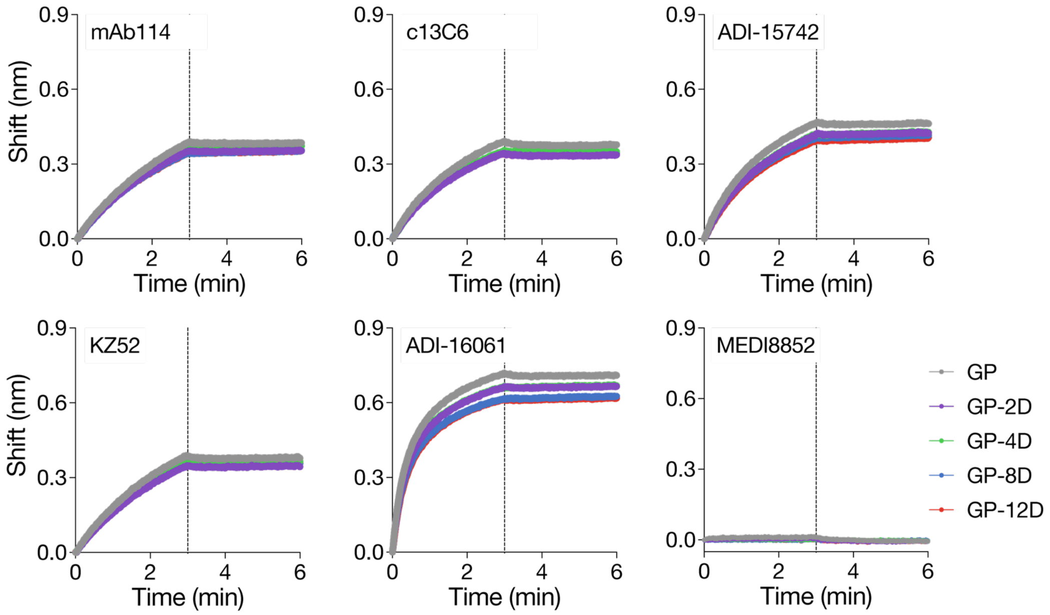

Supplementary Fig. 1 | Antigenicity of oligoD-modified GP. BLI binding profiles of wild-type or oligoD-modified GP with five GP-specific mAbs (mAb114, c13C6, ADI-15742, KZ52 and ADI-16061). An HA-specific mAb (MEDI8852) served as a negative control. Vertical dashed lines indicate the beginning of dissociation.

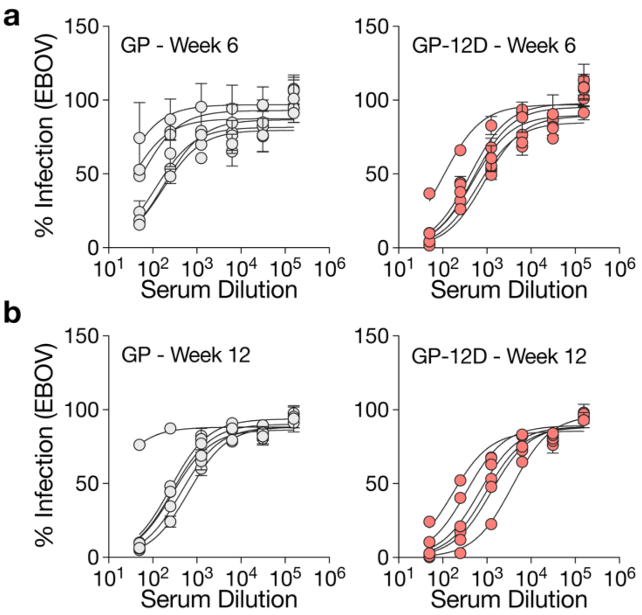

Supplementary Fig. 2 | Analysis of serological responses against GP or GP-12D.

Neutralization of Ebola GP-pseudotyped lentiviruses by antisera collected at six (**a**) or 12 weeks (**b**) post-immunization (*n*=6 mice per group). Each curve is derived from a single mouse. Data are presented as mean ± s.d. of technical duplicates.

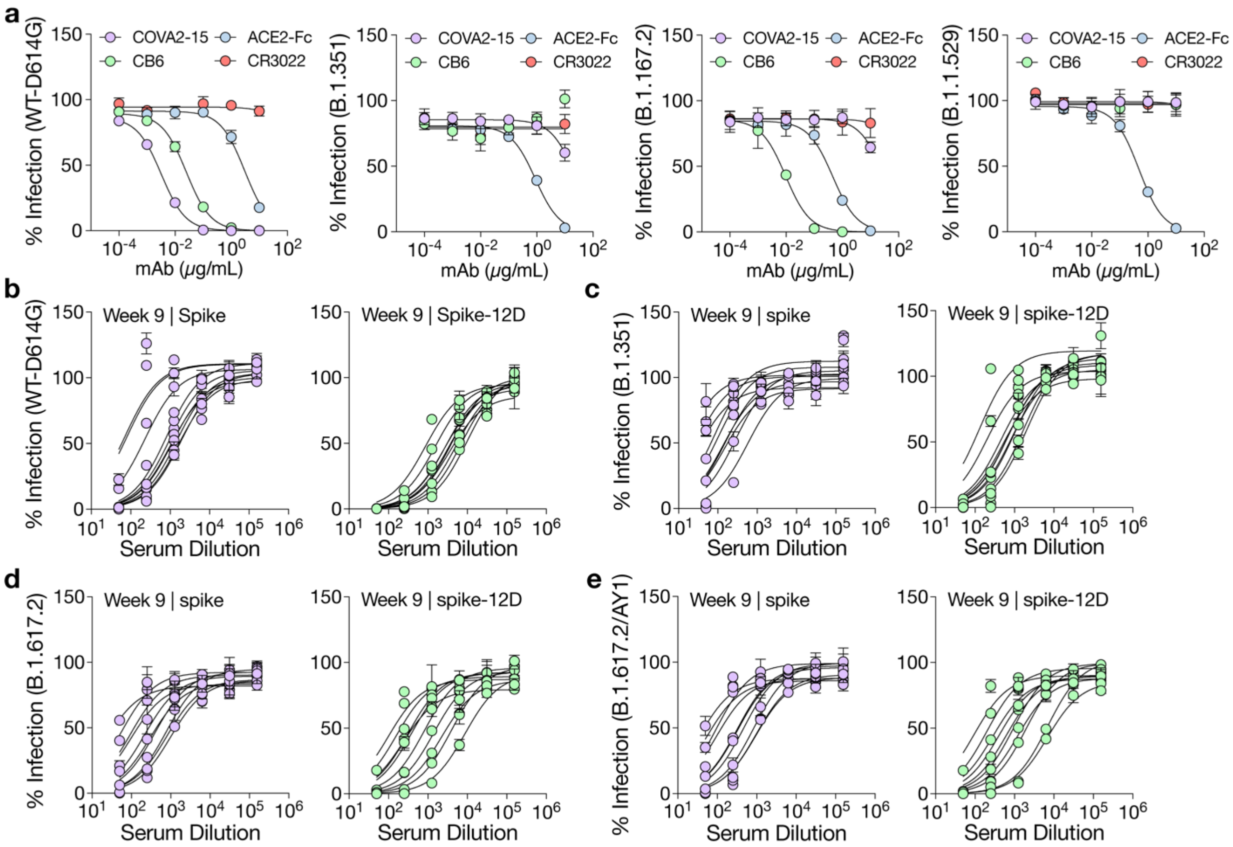

Supplementary Fig. 3 | Analysis of serological responses against spike or spike-12D. a, Validation of the neutralization assay with ACE2-Fc and three Spike-specific mAbs (COVA-215, CB6 and CR3022) against spike-pseudotyped lentiviruses and three variants of concerns (B.1.351, B.1.167.2 and B.1.1.529). Data are presented as mean ± s.d. (*n*=4 technical replicates). **b-e**, Serum neutralization of wild-type SARS-CoV-2 pseudoviruses (**b**) and variants of concern, including B.1.351 (**c**), B.1.617.2 (**d**) and B.1.617.2/AY1 (**e**) (*n*=10 mice per group). Data are presented as mean ± s.d. of technical duplicates in neutralization curves.

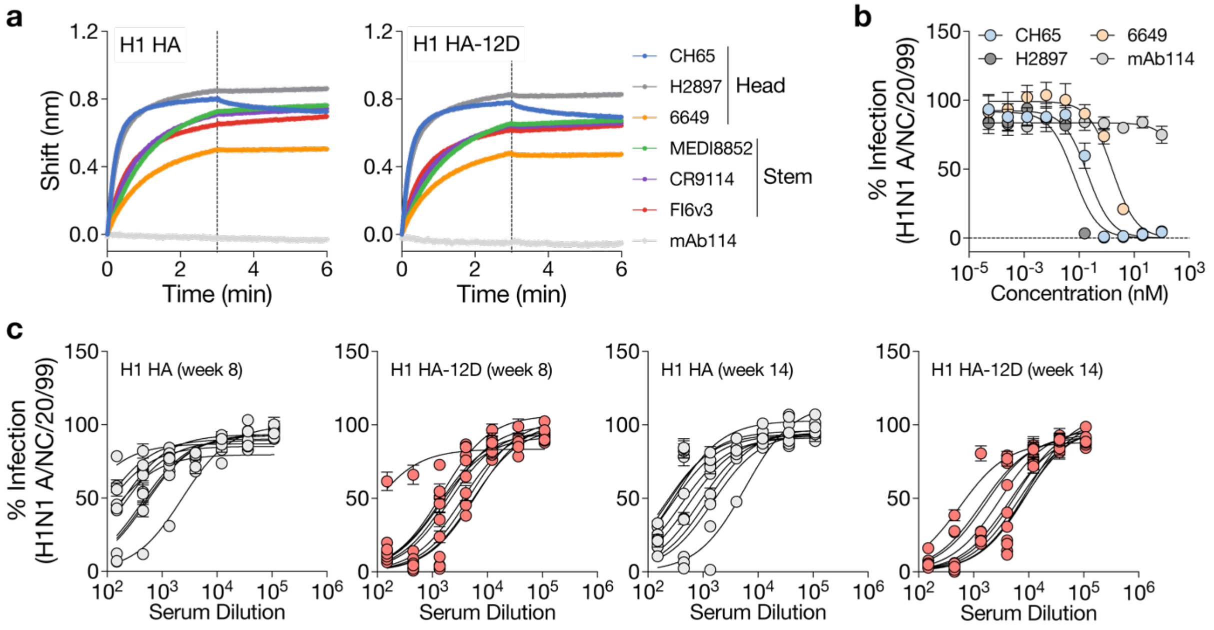

Supplementary Fig. 4 | Antigenicity of and serological analysis against H1 HA and H1 HA-12D. a, BLI binding profiles of wild-type or oligoD-modified H1 HA with three HA-head-directed mAbs (CH65, H2897 and 6649) and three HA-stem-directed mAbs (MEDI8852, CR9114 and FI6v3). A GP-specific mAb (mAb114) served as a negative control. Vertical dashed lines indicate the beginning of dissociation. **b**, Validation of the Influenza A virus (IAV) microneutralization assay with three H1 HA-specific mAbs (CH65, H2897 and 6649) against authentic A/New Caledonia/20/1999 (H1N1 A/NC/20/99) viruses. The dashed line indicates 100% viral neutralization by mAbs. Data are presented as mean ± s.d. (n=4 technical replicates). **c**, Neutralization of H1N1 A/NC/20/99 viruses by antisera collected at weeks eight or 14 post-immunization (*n*=10 mice per group). Each curve is derived from a single mouse. Data are presented as mean ± s.d. of technical duplicates in neutralization curves.

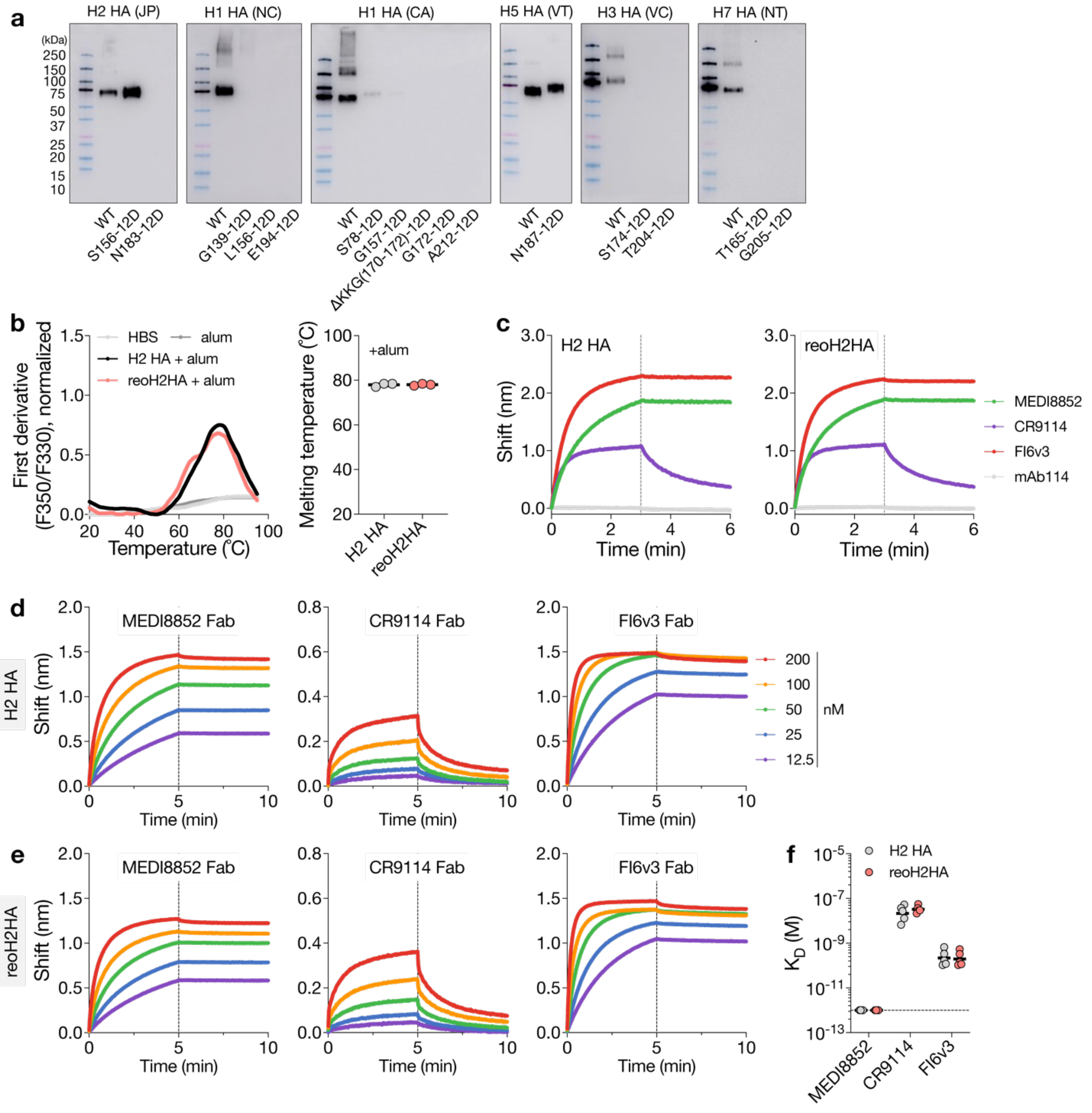

Supplementary Fig. 5 | OligoD insertion into HA-head. a, Screening of oligoD insertion to the head region of different HAs by Western-blot analysis. Blots were detected by a mouse anti-His Tag antibody. HA sequences were from H1 HA (NC – A/New Caledonia/20/99), H1 HA (CA – A/California/7/09), H2 HA (JP – A/Japan/305/1957), H5 HA (VT – A/Vietnam/1203/2004), H3 HA (VC – A/Victoria/3/1975) or H7 HA (NT – A/FPV/Dutch/1927). **b**, Thermal melting profiles and T_m of H2 HA and reoH2HA in the presence of alum (HA: alum = 1: 10, *w/w*). Data are

presented as mean ± s.d. in the dot plots (n=3 samples per group). **c**, BLI binding profiles of H2 HA or reoH2HA with three HA-stem-directed mAbs (MEDI8852, CR9114 and FI6v3). A GP-specific mAb (mAb114) served as a negative control. Vertical dashed lines indicate the beginning of dissociation. **d,e**, BLI binding profiles of H2 HA (**d**) or reoH2HA (**e**) with antigen-binding fragments (Fabs) of MEDI8852, CR9114 or FI6v3. Dashed lines indicate the beginning of dissociation. **f**, Binding affinities (K_D) of stem-directed antibodies to H2 HA or reoH2HA calculated based on individual curves in **d,e**. Data are presented as the geometric mean of the log_{10}-transformed K_D values. The dashed line indicates the limit of quantification (10^{-12} M^{-1}).

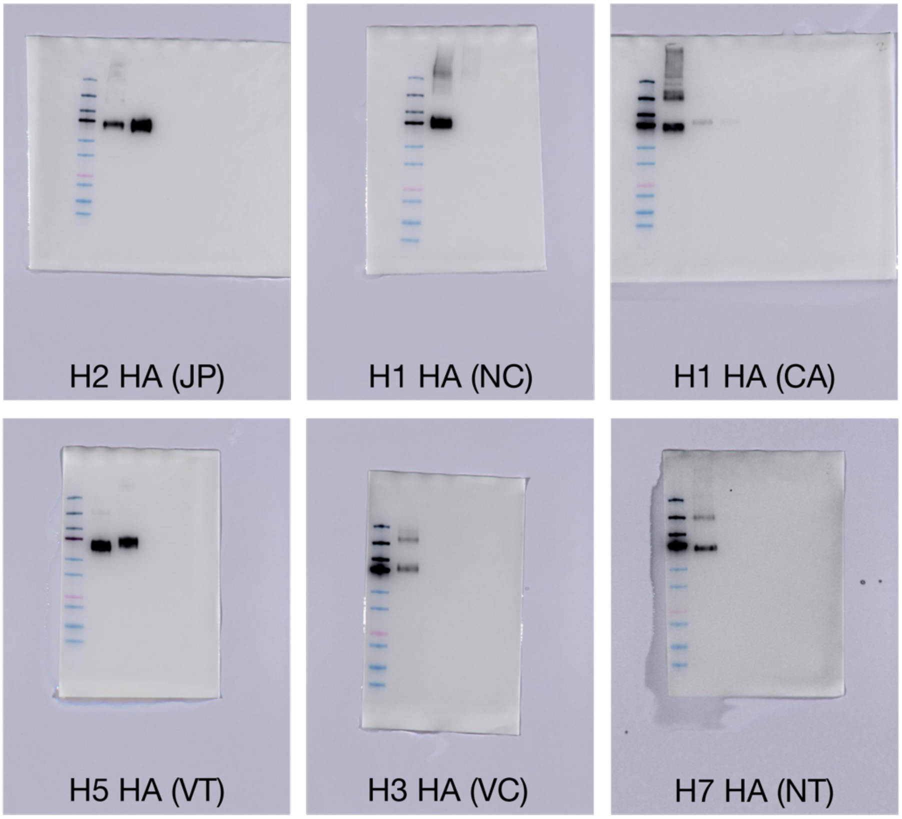

Source data for Supplementary Fig. 5a.

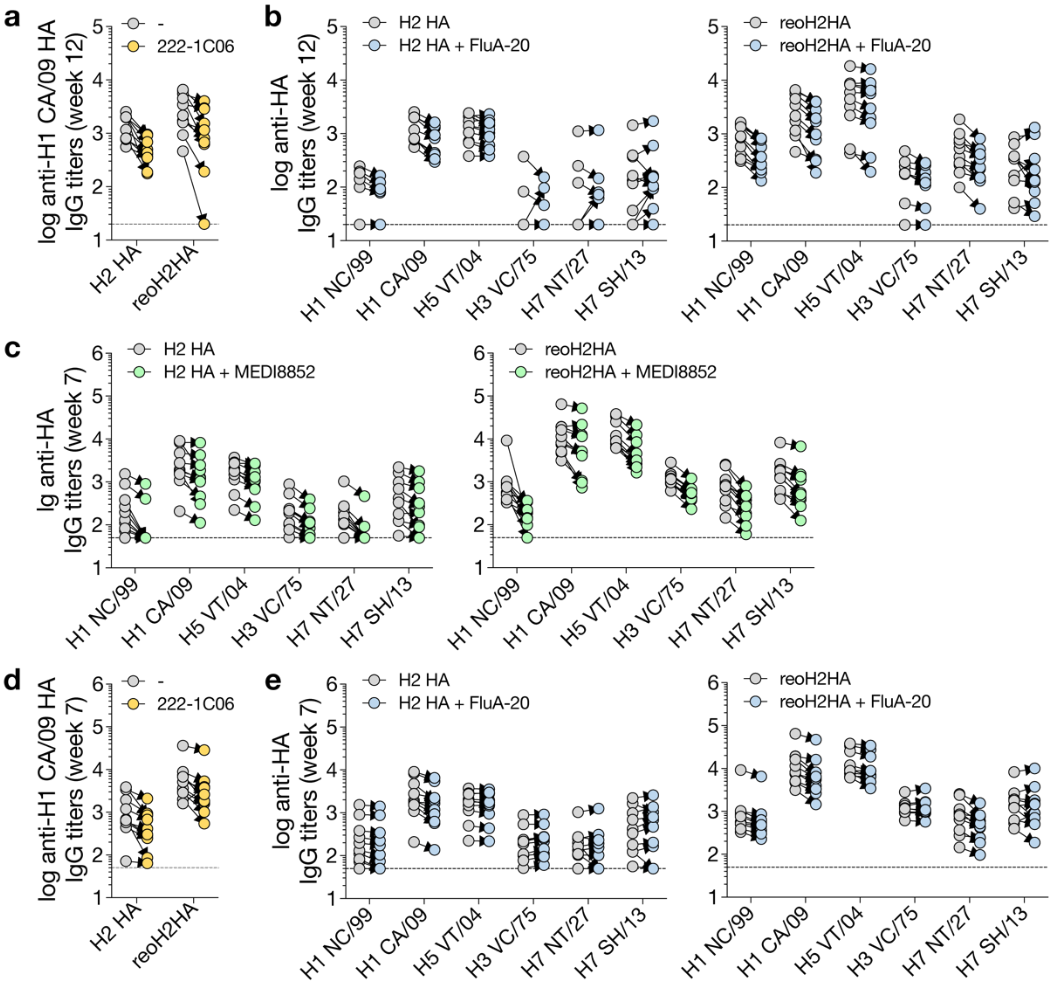

Supplementary Fig. 6 | Competition ELISA. a,b, Analysis with week-12 antisera from the immunization study in **Fig. 5e**. Serum binding titers to H1 HA (CA/09) in the presence of 222-1C06 (**a**), or to different group 1 and group 2 HAs in the presence of FluA-20 (**b**). Each circle represents a single mouse (*n*=10). **c-e**, Analysis with week-7 antisera from the immunization study in **Fig. 6a**. Serum binding titers to group 1 and 2 HAs in the presence of MEDI8852 (**c**), to H1 HA (CA/09) in the presence of a 222-1C06 (**d**), or to different group 1 and group 2 HAs in the presence of FluA-20 (**e**). Dashed lines indicate the limit of quantification. Data are presented as the geometric mean ± s.d. of the log_{10}-transformed values.

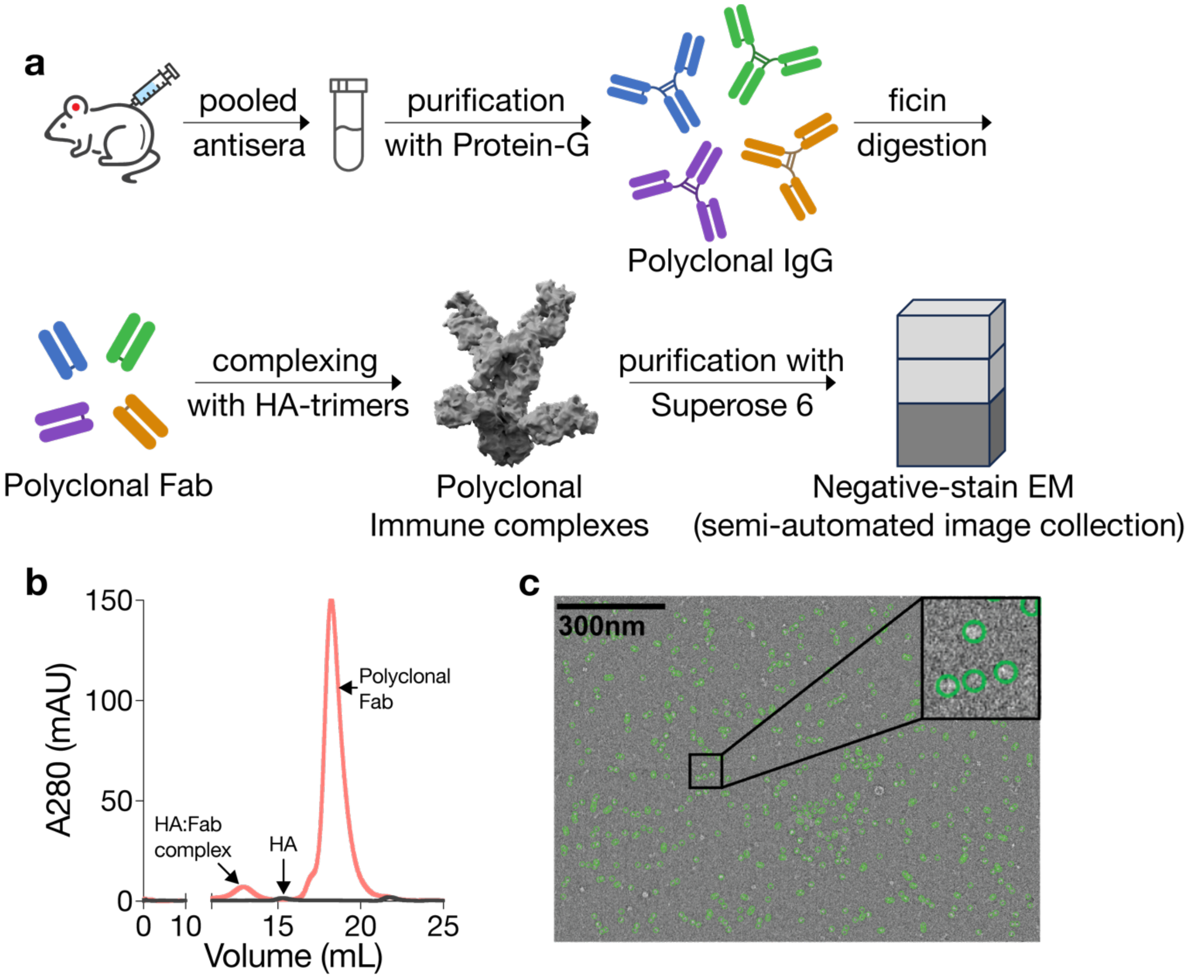

Supplementary Fig. 7 | Negative-stain electron microscopy polyclonal epitope mapping (nsEMPEM). a, Workflow of nsEMPEM. Polyclonal IgGs were purified from pooled mouse antisera followed by ficin digestion to antigen-binding fragments (Fabs). Resulting Fabs were complexed with different HA-trimers and purified with size-exclusion chromatography. Polyclonal immune complexes were then imaged in a semi-automated fashion by negative-stain electron microscopy. **b**, Purification of polyclonal immune complexes by size-exclusion chromatography. **c**, A representative nsEM micrograph. Singles particles are highlighted in the top right box. Scale bar, 300 nm.

Supplementary Table 1 | Molecular weight calculation from SEC-MALS analysis.

Proteins	Theoretical Mw* (×10^5 Da)	Mw from SEC-MALS (×10^5 Da)
GP	1.7	2.0 ± (9.0%)
GP-12D	1.8	2.1 ± (7.6%)
spike	4.0	4.1 ± (9.4%)
spike-12D	4.0	4.4 ± (9.2%)
H1 HA	1.9	1.9 ± (7.7%)
H1 HA-12D	1.9	1.9 ± (9.5%)
H2 HA	1.9	1.9 ± (10.4%)
reoH2HA	1.9	2.0 ± (7.6%)

*Glycosylation was not accounted for in theoretical Mw calculation.
Mw is calculated by the ASTRA software and reported as Mw ± (% s.d.).

Supplementary Notes | Sequences of antigens

• All sequences are from N-terminus to C-terminus

• Signal peptides on the N-terminus are marked in grey

>GP

[1]MGVTGILQLPRDRFKRTSFFLWVIILFQRTFSIPLGVIHNSTLQVSDVDKLVCRDKLSSTN

QLRSVGLNLEGNGVATDVPSATKRWGFRSGVPPKVVNYEAGEWAENCYNLEIKKPDG

SECLPAAPDGIRGFPRCRYVHKVSGTGPCAGDFAFHKEGAFFLYDRLASTVIYRGTTFAE

GVVAFLILPQAKKDFFSSHPLREPVNATEDPSSGYYSTTIRYQATGFGTNETEYLFEVDN

LTYVQLESRFTPQFLLQLNETIYTSGKRSNTTGKLIWKVNPEIDTTIGEWAFWETKKNLT

RKIRSEELSFAGLITGGRRTRREAIVNAQPKCNPNLHYWTTQDEGAAIGLAWIPYFGPAA

EGIYIEGLMHNQDGLICGLRQLANETTQALQLFLRATTELRTFSILNRKAIDFLLQRWGG

TCHILGPDCCIEPHDWTKNITDKIDQIIHDFVDKTLPDQGDNDNWWTGWRQWIPAGI-
MKQIEDKIEEILSKIYHIENEIARIKKLIGEVASSS-GLNDIFEAQKIEWHE-AHHHHHHG

>GP-2D

[1]MGVTGILQLPRDRFKRTSFFLWVIILFQRTFSIPLGVIHNSTLQVSDVDKLVCRDKLSSTN
QLRSVGLNLEGNGVATDVPSATKRWGFRSGVPPKVVNYEAGEWAENCYNLEIKKPDG
SECLPAAPDGIRGFPRCRYVHKVSGTGPCAGDFAFHKEGAFFLYDRLASTVIYRGTTFAE
GVVAFLILPQAKKDFFSSHPLREPVNATEDPSSGYYSTTIRYQATGFGTNETEYLFEVDN
LTYVQLESRFTPQFLLQLNETIYTSGKRSNTTGKLIWKVNPEIDTTIGEWAFWETKKNLT
RKIRSEELSFAGLITGGRRTRREAIVNAQPKCNPNLHYWTTQDEGAAIGLAWIPYFGPAA
EGIYIEGLMHNQDGLICGLRQLANETTQALQLFLRATTELRTFSILNRKAIDFLLQRWGG
TCHILGPDCCIEPHDWTKNITDKIDQIIHDFVDKTLPDQGDNDNWWTGWRQWIPAGI-
MKQIEDKIEEILSKIYHIENEIARIKKLIGEVASSS-GLNDIFEAQKIEWHE-AHHHHHHG-
GSDD

>GP-4D

[1]MGVTGILQLPRDRFKRTSFFLWVIILFQRTFSIPLGVIHNSTLQVSDVDKLVCRDKLSSTN
QLRSVGLNLEGNGVATDVPSATKRWGFRSGVPPKVVNYEAGEWAENCYNLEIKKPDG
SECLPAAPDGIRGFPRCRYVHKVSGTGPCAGDFAFHKEGAFFLYDRLASTVIYRGTTFAE
GVVAFLILPQAKKDFFSSHPLREPVNATEDPSSGYYSTTIRYQATGFGTNETEYLFEVDN
LTYVQLESRFTPQFLLQLNETIYTSGKRSNTTGKLIWKVNPEIDTTIGEWAFWETKKNLT
RKIRSEELSFAGLITGGRRTRREAIVNAQPKCNPNLHYWTTQDEGAAIGLAWIPYFGPAA
EGIYIEGLMHNQDGLICGLRQLANETTQALQLFLRATTELRTFSILNRKAIDFLLQRWGG

TCHILGPDCCIEPHDWTKNITDKIDQIIHDFVDKTLPDQGDNDNWWTGWRQWIPAGI-MKQIEDKIEEILSKIYHIENEIARIKKLIGEVASSS-GLNDIFEAQKIEWHE-AHHHHHHG-**<u>GSDDDD</u>**

>GP-8D

[1]MGVTGILQLPRDRFKRTSFFLWVIILFQRTFSIPLGVIHNSTLQVSDVDKLVCRDKLSSTN
QLRSVGLNLEGNGVATDVPSATKRWGFRSGVPPKVVNYEAGEWAENCYNLEIKKPDG
SECLPAAPDGIRGFPRCRYVHKVSGTGPCAGDFAFHKEGAFFLYDRLASTVIYRGTTFAE
GVVAFLILPQAKKDFFSSHPLREPVNATEDPSSGYYSTTIRYQATGFGTNETEYLFEVDN
LTYVQLESRFTPQFLLQLNETIYTSGKRSNTTGKLIWKVNPEIDTTIGEWAFWETKKNLT
RKIRSEELSFAGLITGGRRTRREAIVNAQPKCNPNLHYWTTQDEGAAIGLAWIPYFGPAA
EGIYIEGLMHNQDGLICGLRQLANETTQALQLFLRATTELRTFSILNRKAIDFLLQRWGG
TCHILGPDCCIEPHDWTKNITDKIDQIIHDFVDKTLPDQGDNDNWWTGWRQWIPAGI-MKQIEDKIEEILSKIYHIENEIARIKKLIGEVASSS-GLNDIFEAQKIEWHE-AHHHHHHG-**<u>GSDDDDDDDD</u>**

>GP-12D

[1]MGVTGILQLPRDRFKRTSFFLWVIILFQRTFSIPLGVIHNSTLQVSDVDKLVCRDKLSSTN
QLRSVGLNLEGNGVATDVPSATKRWGFRSGVPPKVVNYEAGEWAENCYNLEIKKPDG
SECLPAAPDGIRGFPRCRYVHKVSGTGPCAGDFAFHKEGAFFLYDRLASTVIYRGTTFAE
GVVAFLILPQAKKDFFSSHPLREPVNATEDPSSGYYSTTIRYQATGFGTNETEYLFEVDN
LTYVQLESRFTPQFLLQLNETIYTSGKRSNTTGKLIWKVNPEIDTTIGEWAFWETKKNLT
RKIRSEELSFAGLITGGRRTRREAIVNAQPKCNPNLHYWTTQDEGAAIGLAWIPYFGPAA

EGIYIEGLMHNQDGLICGLRQLANETTQALQLFLRATTELRTFSILNRKAIDFLLQRWGG
TCHILGPDCCIEPHDWTKNITDKIDQIIHDFVDKTLPDQGDNDNWWTGWRQWIPAGI-
MKQIEDKIEEILSKIYHIENEIARIKKLIGEVASSS-GLNDIFEAQKIEWHE-AHHHHHHG-
GSDDDDDDDDDDDD

>GP-R200-8D

[1]MGVTGILQLPRDRFKRTSFFLWVIILFQRTFSIPLGVIHNSTLQVSDVDKLVCRDKLSSTN
QLRSVGLNLEGNGVATDVPSATKRWGFRSGVPPKVVNYEAGEWAENCYNLEIKKPDG
SECLPAAPDGIRGFPRCRYVHKVSGTGPCAGDFAFHKEGAFFLYDRLASTVIYRGTTFAE
GVVAFLILPQAKKDFFSSHPLR**DDDDDDDD**EPVNATEDPSSGYYSTTIRYQATGFGTNET
EYLFEVDNLTYVQLESRFTPQFLLQLNETIYTSGKRSNTTGKLIWKVNPEIDTTIGEWAF
WETKKNLTRKIRSEELSFAGLITGGRRTRREAIVNAQPKCNPNLHYWTTQDEGAAIGLA
WIPYFGPAAEGIYIEGLMHNQDGLICGLRQLANETTQALQLFLRATTELRTFSILNRKAID
FLLQRWGGTCHILGPDCCIEPHDWTKNITDKIDQIIHDFVDKTLPDQGDNDNWWTGWR
QWIPAGI-MKQIEDKIEEILSKIYHIENEIARIKKLIGEVASSS-GLNDIFEAQKIEWHE-
AHHHHHHG

>GP-R200-12D

[1]MGVTGILQLPRDRFKRTSFFLWVIILFQRTFSIPLGVIHNSTLQVSDVDKLVCRDKLSSTN
QLRSVGLNLEGNGVATDVPSATKRWGFRSGVPPKVVNYEAGEWAENCYNLEIKKPDG
SECLPAAPDGIRGFPRCRYVHKVSGTGPCAGDFAFHKEGAFFLYDRLASTVIYRGTTFAE
GVVAFLILPQAKKDFFSSHPLR**DDDDDDDDDDDD**EPVNATEDPSSGYYSTTIRYQATGF
GTNETEYLFEVDNLTYVQLESRFTPQFLLQLNETIYTSGKRSNTTGKLIWKVNPEIDTTIG

EWAFWETKKNLTRKIRSEELSFAGLITGGRRTRREAIVNAQPKCNPNLHYWTTQDEGAA
IGLAWIPYFGPAAEGIYIEGLMHNQDGLICGLRQLANETTQALQLFLRATTELRTFSILNR
KAIDFLLQRWGGTCHILGPDCCIEPHDWTKNITDKIDQIIHDFVDKTLPDQGDNDNWWT
GWRQWIPAGI-MKQIEDKIEEILSKIYHIENEIARIKKLIGEVASSS-GLNDIFEAQKIEWHE-
AHHHHHHG

>GP-T294-8D

[1]MGVTGILQLPRDRFKRTSFFLWVIILFQRTFSIPLGVIHNSTLQVSDVDKLVCRDKLSSTN
QLRSVGLNLEGNGVATDVPSATKRWGFRSGVPPKVVNYEAGEWAENCYNLEIKKPDG
SECLPAAPDGIRGFPRCRYVHKVSGTGPCAGDFAFHKEGAFFLYDRLASTVIYRGTTFAE
GVVAFLILPQAKKDFFSSHPLREPVNATEDPSSGYYSTTIRYQATGFGTNETEYLFEVDN
LTYVQLESRFTPQFLLQLNETIYTSGKRSNTTGKLIWKVNPEIDTTIGEWAFWET**DDDDD
DDD**KKNLTRKIRSEELSFAGLITGGRRTRREAIVNAQPKCNPNLHYWTTQDEGAAIGLA
WIPYFGPAAEGIYIEGLMHNQDGLICGLRQLANETTQALQLFLRATTELRTFSILNRKAID
FLLQRWGGTCHILGPDCCIEPHDWTKNITDKIDQIIHDFVDKTLPDQGDNDNWWTGWR
QWIPAGI-MKQIEDKIEEILSKIYHIENEIARIKKLIGEVASSS-GLNDIFEAQKIEWHE-
AHHHHHHG

>GP-T294-12D

[1]MGVTGILQLPRDRFKRTSFFLWVIILFQRTFSIPLGVIHNSTLQVSDVDKLVCRDKLSSTN
QLRSVGLNLEGNGVATDVPSATKRWGFRSGVPPKVVNYEAGEWAENCYNLEIKKPDG
SECLPAAPDGIRGFPRCRYVHKVSGTGPCAGDFAFHKEGAFFLYDRLASTVIYRGTTFAE
GVVAFLILPQAKKDFFSSHPLREPVNATEDPSSGYYSTTIRYQATGFGTNETEYLFEVDN

LTYVQLESRFTPQFLLQLNETIYTSGKRSNTTGKLIWKVNPEIDTTIGEWAFWET**DDDDD**
DDDDDDDKKNLTRKIRSEELSFAGLITGGRRTRREAIVNAQPKCNPNLHYWTTQDEGAA
IGLAWIPYFGPAAEGIYIEGLMHNQDGLICGLRQLANETTQALQLFLRATTELRTFSILNR
KAIDFLLQRWGGTCHILGPDCCIEPHDWTKNITDKIDQIIHDFVDKTLPDQGDNDNWWT
GWRQWIPAGI-MKQIEDKIEEILSKIYHIENEIARIKKLIGEVASSS-GLNDIFEAQKIEWHE-
AHHHHHHG

>GP-A309-8D

[1]MGVTGILQLPRDRFKRTSFFLWVIILFQRTFSIPLGVIHNSTLQVSDVDKLVCRDKLSSTN
QLRSVGLNLEGNGVATDVPSATKRWGFRSGVPPKVVNYEAGEWAENCYNLEIKKPDG
SECLPAAPDGIRGFPRCRYVHKVSGTGPCAGDFAFHKEGAFFLYDRLASTVIYRGTTFAE
GVVAFLILPQAKKDFFSSHPLREPVNATEDPSSGYYSTTIRYQATGFGTNETEYLFEVDN
LTYVQLESRFTPQFLLQLNETIYTSGKRSNTTGKLIWKVNPEIDTTIGEWAFWETKKNLT
RKIRSEELSFA**DDDDDDDD**GLITGGRRTRREAIVNAQPKCNPNLHYWTTQDEGAAIGLA
WIPYFGPAAEGIYIEGLMHNQDGLICGLRQLANETTQALQLFLRATTELRTFSILNRKAID
FLLQRWGGTCHILGPDCCIEPHDWTKNITDKIDQIIHDFVDKTLPDQGDNDNWWTGWR
QWIPAGI-MKQIEDKIEEILSKIYHIENEIARIKKLIGEVASSS-GLNDIFEAQKIEWHE-
AHHHHHHG

>GP-A309-12D

[1]MGVTGILQLPRDRFKRTSFFLWVIILFQRTFSIPLGVIHNSTLQVSDVDKLVCRDKLSSTN
QLRSVGLNLEGNGVATDVPSATKRWGFRSGVPPKVVNYEAGEWAENCYNLEIKKPDG
SECLPAAPDGIRGFPRCRYVHKVSGTGPCAGDFAFHKEGAFFLYDRLASTVIYRGTTFAE

GVVAFLILPQAKKDFFSSHPLREPVNATEDPSSGYYSTTIRYQATGFGTNETEYLFEVDN
LTYVQLESRFTPQFLLQLNETIYTSGKRSNTTGKLIWKVNPEIDTTIGEWAFWETKKNLT
RKIRSEELSFA**DDDDDDDDDDDD**GLITGGRRTRREAIVNAQPKCNPNLHYWTTQDEGA
AIGLAWIPYFGPAAEGIYIEGLMHNQDGLICGLRQLANETTQALQLFLRATTELRTFSILN
RKAIDFLLQRWGGTCHILGPDCCIEPHDWTKNITDKIDQIIHDFVDKTLPDQGDNDNWW
TGWRQWIPAGI-MKQIEDKIEEILSKIYHIENEIARIKKLIGEVASSS-
GLNDIFEAQKIEWHE-AHHHHHHG

>spike

[1]MFVFLVLLPLVSSQCVNLTTRTQLPPAYTNSFTRGVYYPDKVFRSSVLHSTQDLFLPFFS
NVTWFHAIHVSGTNGTKRFDNPVLPFNDGVYFASTEKSNIIRGWIFGTTLDSKTQSLLIV
NNATNVVIKVCEFQFCNDPFLGVYYHKNNKSWMESEFRVYSSANNCTFEYVSQPFLMD
LEGKQGNFKNLREFVFKNIDGYFKIYSKHTPINLVRDLPQGFSALEPLVDLPIGINITRFQT
LLALHRSYLTPGDSSSGWTAGAAAYYVGYLQPRTFLLKYNENGTITDAVDCALDPLSET
KCTLKSFTVEKGIYQTSNFRVQPTESIVRFPNITNLCPFGEVFNATRFASVYAWNRKRISN
CVADYSVLYNSASFSTFKCYGVSPTKLNDLCFTNVYADSFVIRGDEVRQIAPGQTGKIA
DYNYKLPDDFTGCVIAWNSNNLDSKVGGNYNYLYRLFRKSNLKPFERDISTEIYQAGST
PCNGVEGFNCYFPLQSYGFQPTNGVGYQPYRVVVLSFELLHAPATVCGPKKSTNLVKN
KCVNFNFNGLTGTGVLTESNKKFLPFQQFGRDIADTTDAVRDPQTLEILDITPCSFGGVS
VITPGTNTSNQVAVLYQDVNCTEVPVAIHADQLTPTWRVYSTGSNVFQTRAGCLIGAEH
VNNSYECDIPIGAGICASYQTQTNSPASVASQSIIAYTMSLGAENSVAYSNNSIAIPTNFTI
SVTTEILPVSMTKTSVDCTMYICGDSTECSNLLLQYGSFCTQLNRALTGIAVEQDKNTQE
VFAQVKQIYKTPPIKDFGGFNFSQILPDPSKPSKRSFIEDLLFNKVTLADAGFIKQYGDCL

GDIAARDLICAQKFNGLTVLPPLLTDEMIAQYTSALLAGTITSGWTFGAGAALQIPFAMQ
MAYRFNGIGVTQNVLYENQKLIANQFNSAIGKIQDSLSSTASALGKLQDVVNQNAQALN
TLVKQLSSNFGAISSVLNDILSRLDPPEAEVQIDRLITGRLQSLQTYVTQQLIRAAEIRASA
NLAATKMSECVLGQSKRVDFCGKGYHLMSFPQSAPHGVVFLHVTYVPAQEKNFTTAPA
ICHDGKAHFPREGVFVSNGTHWFVTQRNFYEPQIITTDNTFVSGNCDVVIGIVNNTVYDP
LQPELD-MKQIEDKIEEILSKIYHIENEIARIKKLIGEVASSS-GLNDIFEAQKIEWHE-
AHHHHHHG

>spike-12D

[1]MFVFLVLLPLVSSQCVNLTTRTQLPPAYTNSFTRGVYYPDKVFRSSVLHSTQDLFLPFFS
NVTWFHAIHVSGTNGTKRFDNPVLPFNDGVYFASTEKSNIIRGWIFGTTLDSKTQSLLIV
NNATNVVIKVCEFQFCNDPFLGVYYHKNNKSWMESEFRVYSSANNCTFEYVSQPFLMD
LEGKQGNFKNLREFVFKNIDGYFKIYSKHTPINLVRDLPQGFSALEPLVDLPIGINITRFQT
LLALHRSYLTPGDSSSGWTAGAAAYYVGYLQPRTFLLKYNENGTITDAVDCALDPLSET
KCTLKSFTVEKGIYQTSNFRVQPTESIVRFPNITNLCPFGEVFNATRFASVYAWNRKRISN
CVADYSVLYNSASFSTFKCYGVSPTKLNDLCFTNVYADSFVIRGDEVRQIAPGQTGKIA
DYNYKLPDDFTGCVIAWNSNNLDSKVGGNYNYLYRLFRKSNLKPFERDISTEIYQAGST
PCNGVEGFNCYFPLQSYGFQPTNGVGYQPYRVVVLSFELLHAPATVCGPKKSTNLVKN
KCVNFNFNGLTGTGVLTESNKKFLPFQQFGRDIADTTDAVRDPQTLEILDITPCSFGGVS
VITPGTNTSNQVAVLYQDVNCTEVPVAIHADQLTPTWRVYSTGSNVFQTRAGCLIGAEH
VNNSYECDIPIGAGICASYQTQTNSPASVASQSIIAYTMSLGAENSVAYSNNSIAIPTNFTI
SVTTEILPVSMTKTSVDCTMYICGDSTECSNLLLQYGSFCTQLNRALTGIAVEQDKNTQE
VFAQVKQIYKTPPIKDFGGFNFSQILPDPSKPSKRSFIEDLLFNKVTLADAGFIKQYGDCL

GDIAARDLICAQKFNGLTVLPPLLTDEMIAQYTSALLAGTITSGWTFGAGAALQIPFAMQ

MAYRFNGIGVTQNVLYENQKLIANQFNSAIGKIQDSLSSTASALGKLQDVVNQNAQALN

TLVKQLSSNFGAISSVLNDILSRLDPPEAEVQIDRLITGRLQSLQTYVTQQLIRAAEIRASA

NLAATKMSECVLGQSKRVDFCGKGYHLMSFPQSAPHGVVFLHVTYVPAQEKNFTTAPA

ICHDGKAHFPREGVFVSNGTHWFVTQRNFYEPQIITTDNTFVSGNCDVVIGIVNNTVYDP

LQPELD-MKQIEDKIEEILSKIYHIENEIARIKKLIGEVASSS-GLNDIFEAQKIEWHE-

AHHHHHHG-**<u>GGSDDDDDDDDDDDD</u>**

>H1 HA

MYRMQLLSCIALSLALVTNS[1]DTICIGYHANNSTDTVDTVLEKNVTVTHSVNLLEDSHN

GKLCLLKGIAPLQLGNCSVAGWILGNPECELLISKESWSYIVETPNPENGTCFPGYFADY

EELREQLSSVSSFERFEIFPKESSWPNHTVTGVSASCSHNGKSSFYRNLLWLTGKNGLYP

NLSKSYVNNKEKEVLVLWGVHHPPNIGNQRALYHTENAYVSVVSSHYSRRFTPEIAKRP

KVRDQEGRINYYWTLLEPGDTIIFEANGNLIAPWYAFALSRGFGSGIITSNAPMDECDAK

CQTPQGAINSSLPFQNVHPVTIGECPKYVRSAKLRMVTGLRNIPQRETGGLFGAIAGFIEG

GWTGMVDGWYGYHHQNEQGSGYAADQKSTQNAINGITNKVNSVIEKMNTQFTAVGK

EFNKLERRMENLNKKVDDGFLDIWTYNAELLVLLENERTLDFHDSNVKNLYEKVKSQL

KNNAKEIGNGCFEFYHKCNNECMESVKNGTYDYPKYSEESKLNREKIDGS-

GYIPEAPRDGQAYVRKDGEWVLLSTFLGS-GLNDIFEAQKIEWHE-GHHHHHH

>H1 HA-12D

MYRMQLLSCIALSLALVTNS[1]DTICIGYHANNSTDTVDTVLEKNVTVTHSVNLLEDSHN

GKLCLLKGIAPLQLGNCSVAGWILGNPECELLISKESWSYIVETPNPENGTCFPGYFADY

EELREQLSSVSSFERFEIFPKESSWPNHTVTGVSASCSHNGKSSFYRNLLWLTGKNGLYP

NLSKSYVNNKEKEVLVLWGVHHPPNIGNQRALYHTENAYVSVVSSHYSRRFTPEIAKRP

KVRDQEGRINYYWTLLEPGDTIIFEANGNLIAPWYAFALSRGFGSGIITSNAPMDECDAK

CQTPQGAINSSLPFQNVHPVTIGECPKYVRSAKLRMVTGLRNIPQRETGGLFGAIAGFIEG

GWTGMVDGWYGYHHQNEQGSGYAADQKSTQNAINGITNKVNSVIEKMNTQFTAVGK

EFNKLERRMENLNKKVDDGFLDIWTYNAELLVLLENERTLDFHDSNVKNLYEKVKSQL

KNNAKEIGNGCFEFYHKCNNECMESVKNGTYDYPKYSEESKLNREKIDGS-

GYIPEAPRDGQAYVRKDGEWVLLSTFLGS-GLNDIFEAQKIEWHE-GHHHHHH-

GSDDDDDDDDDDDD

>H7 HA-12D

MNTQILVFALIAIIPTNAD[1]KICLGHHAVSNGTKVNTLTERGVEVVNATETVERTNIPRICS

KGKRTVDLGQCGLLGTITGPPQCDQFLEFSADLIIERREGSDVCFPGKFVNEEALRQILRE

SGGIDKEAMGFTYSGIRTNGATSACRRSGSSFYAEMKWLLSNTDNAAFPQMTKSYKNT

RKSPALIVWGIHHSVSTAEQTKLYGSGNKLVTVGSSNYQQSFVPSPGARPQVNGLSGRI

DFHWLMLNPNDTVTFSFNGAFIAPDRASFLRGKSMGIQSGVQVDANCEGDCYHSGGTII

SNLPFQNIDSRAVGKCPRYVKQRSLLLATGMKNVPEIPKGRGLFGAIAGFIENGWEGLID

GWYGFRHQNAQGEGTAADYKSTQSAIDQITGKLNRLIEKTNQQFELIDNEFNEVEKQIG

NVINWTRDSITEVWSYNAELLVAMENQHTIDLADSEMDKLYERVKRQLRENAEEDGTG

CFEIFHKCDDDCMASIRNNTYDHSKYREEAMQNRIQIDGS-

GYIPEAPRDGQAYVRKDGEWVLLSTFLGS-GLNDIFEAQKIEWHE-GHHHHHH-

GSDDDDDDDDDDDD

>H2 HA

MEKIVLLFAIVSLVKS[1]RGDQICIGYHANNSTEKVDTILERNVTVTHAKDILEKTHNGKLC
KLNGIPPLELGDCSIAGWLLGNPECDRLLSVPEWSYIMEKENPRDGLCFPGSFNDYEELK
YLLSSVKHFEKVKILPKDRWTQHTTTGGSRACAVSGNPSFFRNMVWLTKKGSDYPVAK
GSYNNTSGEQMLIIWGVHHPNDETEQRTLYQNVGTYVSVGTSTLNKRSTPEIATRPKVN
GLGSRMEFSWTLLDMWDTINFESTGNLIAPEYGFKISKRGSSGIMKTEGTLENCETKCQT
PLGAINTTLPFHNVHPLTIGECPRYVKSEKLVLATGLRNVPQIESRGLFGAIAGFIEGGWQ
GMVDGWYGYHHSNDQGSGYAADKESTQKAFDGITNKVNSVIEKMNTQFEAVGKEFSN
LERRLENLNKKMEDGFLDVWTYNAELLVLMENERTLDFHDSNVKNLYDKVRMQLRD
NVKELGNGCFEFYHKCDDECMNSVKNGTYDYPKYEEESKLNRNEGSGYIPEAPRDGQA
YVRKDGEWVLLSTFLGS-GLNDIFEAQKIEWHE-GHHHHHH

>reoH2HA

MEKIVLLFAIVSLVKS[1]RGDQICIGYHANNSTEKVDTILERNVTVTHAKDILEKTHNGKLC
KLNGIPPLELGDCSIAGWLLGNPECDRLLSVPEWSYIMEKENPRDGLCFPGSFNDYEELK
YLLSSVKHFEKVKILPKDRWTQHTTTGGSRACAVSGNPSFFRNMVWLTKKGS**DDDDDD**
DDDDDDDYPVAKGSYNNTSGEQMLIIWGVHHPNDETEQRTLYQNVGTYVSVGTSTLN
KRSTPEIATRPKVNGLGSRMEFSWTLLDMWDTINFESTGNLIAPEYGFKISKRGSSGIMK
TEGTLENCETKCQTPLGAINTTLPFHNVHPLTIGECPRYVKSEKLVLATGLRNVPQIESRG
LFGAIAGFIEGGWQGMVDGWYGYHHSNDQGSGYAADKESTQKAFDGITNKVNSVIEK
MNTQFEAVGKEFSNLERRLENLNKKMEDGFLDVWTYNAELLVLMENERTLDFHDSNV
KNLYDKVRMQLRDNVKELGNGCFEFYHKCDDECMNSVKNGTYDYPKYEEESKLNRN
EGSGYIPEAPRDGQAYVRKDGEWVLLSTFLGS-GLNDIFEAQKIEWHE-GHHHHHH
