# Supplementary material for: Vaccine design via antigen reorientation
Source: Nat Chem Biol. 2024 Jan 15;20(8):1012–21. doi: 10.1038/s41589-023-01529-6 (PMC11247139; doi:10.1038/s41589-023-01529-6)
Supplement: Supplementary file 2 — Reporting Summary [file 41589_2023_1529_MOESM2_ESM.pdf]

nature portfolio

Corresponding author(s): Peter S. Kim

Last updated by author(s): Dec 7, 2023

Reporting Summary

Nature Portfolio wishes to improve the reproducibility of the work that we publish. This form provides structure for consistency and transparency in reporting. For further information on Nature Portfolio policies, see our Editorial Policies and the Editorial Policy Checklist.

Statistics

For all statistical analyses, confirm that the following items are present in the figure legend, table legend, main text, or Methods section.

n/a	Confirmed	
☐	☒	The exact sample size (*n*) for each experimental group/condition, given as a discrete number and unit of measurement
☐	☒	A statement on whether measurements were taken from distinct samples or whether the same sample was measured repeatedly
☐	☒	The statistical test(s) used AND whether they are one- or two-sided<br>*Only common tests should be described solely by name; describe more complex techniques in the Methods section.*
☒	☐	A description of all covariates tested
☐	☒	A description of any assumptions or corrections, such as tests of normality and adjustment for multiple comparisons
☐	☒	A full description of the statistical parameters including central tendency (e.g. means) or other basic estimates (e.g. regression coefficient) AND variation (e.g. standard deviation) or associated estimates of uncertainty (e.g. confidence intervals)
☐	☒	For null hypothesis testing, the test statistic (e.g. *F*, *t*, *r*) with confidence intervals, effect sizes, degrees of freedom and *P* value noted<br>*Give P values as exact values whenever suitable.*
☒	☐	For Bayesian analysis, information on the choice of priors and Markov chain Monte Carlo settings
☒	☐	For hierarchical and complex designs, identification of the appropriate level for tests and full reporting of outcomes
☒	☐	Estimates of effect sizes (e.g. Cohen's *d*, Pearson's *r*), indicating how they were calculated

Our web collection on statistics for biologists contains articles on many of the points above.

Software and code

Policy information about availability of computer code

Data collection	Biolayer interferometry - Octet System Data Analysis Software version (9.0.0.15)<br>Thermal stability characterization - PR.ThermControl (version 2.3.1)<br>ELISA and Neutralization assays - Tecan i-control (version 2.0) or BioTek Gen5 (version 2.09)<br>Flow cytometry - BD FACSDiva (version 8.0.1)<br>Negative-stain electron microscopy polyclonal epitope mapping - SerialEM (version 4.0), RELION (version 4.0.1), cryoSPARC(version 4.3.1)
Data analysis	GraphPad Prism (version 9.3.1), ASTRA (version 7.3.2.21), FlowJo (version 10.4.0), UCSF Chimera X (version 1.5)

For manuscripts utilizing custom algorithms or software that are central to the research but not yet described in published literature, software must be made available to editors and reviewers. We strongly encourage code deposition in a community repository (e.g. GitHub). See the Nature Portfolio guidelines for submitting code & software for further information.

Data

Policy information about availability of data

All manuscripts must include a data availability statement. This statement should provide the following information, where applicable:

- Accession codes, unique identifiers, or web links for publicly available datasets
- A description of any restrictions on data availability
- For clinical datasets or third party data, please ensure that the statement adheres to our policy

All data supporting the results of this study are available within the main text and its supplementary information. Source data are provided with this paper. 3D negative-stain reconstructions for polyclonal Fab:HA complexes have been deposited in the Electron Microscopy Databank (EMDB, http://www.emdataresource.org/) and are available under the following accession codes: EMD-42044, EMD-42046, EMD-42048, EMD-42056, EMD-42058, and EMD-42059.

Human research participants

Policy information about studies involving human research participants and Sex and Gender in Research.

Reporting on sex and gender	n/a
Population characteristics	n/a
Recruitment	n/a
Ethics oversight	n/a

Note that full information on the approval of the study protocol must also be provided in the manuscript.

Field-specific reporting

Please select the one below that is the best fit for your research. If you are not sure, read the appropriate sections before making your selection.

☒ Life sciences ☐ Behavioural & social sciences ☐ Ecological, evolutionary & environmental sciences

For a reference copy of the document with all sections, see nature.com/documents/nr-reporting-summary-flat.pdf

Life sciences study design

All studies must disclose on these points even when the disclosure is negative.

Sample size	No statistical test was used to determine the sample size. Sample sizes were predetermined from pilot experiments and sample sizes of at least 5 mice per group were considered appropriate to evaluate each antigen and to obtain statistically significant results.
Data exclusions	No data were excluded from analysis.
Replication	Experiments were at least repeated twice and attempts at replication were successful.
Randomization	BALB/c were purchased from Jackson Laboratories and randomly grouped for the studies. Randomization of samples was not necessary as comparisons of samples were examined in parallel.
Blinding	Investigators were involved in the overall conduct of the study. Experiments were conducted in an unblinded way. Blinding was not necessary as comparisons of samples were examined in parallel.

Reporting for specific materials, systems and methods

We require information from authors about some types of materials, experimental systems and methods used in many studies. Here, indicate whether each material, system or method listed is relevant to your study. If you are not sure if a list item applies to your research, read the appropriate section before selecting a response.

Materials & experimental systems

n/a	Involved in the study
☐	☒ Antibodies
☐	☒ Eukaryotic cell lines
☒	☐ Palaeontology and archaeology
☐	☒ Animals and other organisms
☒	☐ Clinical data
☒	☐ Dual use research of concern

Methods

n/a	Involved in the study
☒	☐ ChIP-seq
☐	☒ Flow cytometry
☒	☐ MRI-based neuroimaging

Antibodies

Antibodies used

Monoclonal antibodies (mAbs) against Ebola glycoprotein (mAb114, c13C6, ADI-15742, KZ52 and ADI-16061), SARS-CoV-2 spike (COVA2-15, CB6 and CR3022), or influenza hemagglutinin (CH65, H2897, 6649, MEDI8852, CR9114, FI6v3, 8F8, 8M2, 222-1C06 and FluA-20) were expressed in-house in Expi-293F cells via transient transfection. Their sequences were identified from literature and cloned accordingly.

Mouse anti-His Tag antibody (clone J099B12, BioLegend #652502), goat anti-mouse IgG, HRP conjugated (clone Poly4053, BioLegend #405306), goat anti-mouse IgG1-HRP (SouthernBiotech #1070-05), goat anti-mouse IgG2a-HRP (SouthernBiotech #1083-05), goat anti-mouse IgG2b-HRP (SouthernBiotech #1093-05), rabbit anti-human IgG, HRP conjugated (Abcam #ab6759) and goat anti-rabbit IgG, HRP conjugated (Invitrogen #G-21234) were used as detecting antibodies for Western-blot analysis or enzyme-linked immunosorbent assays (ELISAs). Goat anti-human IgG, gold-conjugated (Electron Microscopy Sciences # 25208) was used for immunogold labeling. Mouse anti-influenza A nucleoprotein antibody (clones A1, A3 blend, Sigma-Aldrich #MAB8251) and rabbit anti-influenza A nucleoprotein antibody (clone HL1078, Invitrogen #MA5-42363) were used for influenza A virus microneutralization assays.

For the analysis of germinal center responses, cells were stained with the following antibodies:
Antibody, fluorophore, clone, catalog number, vendor
anti-CD3, A700, 17A2, 100216, BioLegend
anti-CD4, BV650, GK1.5, 100469, BioLegend
anti-CXCR5, BV711, L138D7, 145529, BioLegend
anti-PD1, PE/Dazzle594, 29F.1A12, 135228, BioLegend
anti-CD19, PerCP-Cy5.5, 6D5, 115534, BioLegend
anti-CD95, PECy7, Jo2, 557653, BD
anti-CD38, BUV395, 90, 740245, BD
anti-IgG, PE, A85-1, 550083, BD

Validation

All antibodies against viral proteins were sequence-confirmed across the variable regions. These antibodies were expressed and purified from Expi293-F cells and validated by their binding to viral proteins as shown in the Bio-layer interferometry or ELISA measurements.
Antibodies used in Western blots, ELISAs and flow cytometry were validated in recent publications by coauthors (PMID: 33527087, PMID: 35288714).

Eukaryotic cell lines

Policy information about cell lines and Sex and Gender in Research

Cell line source(s)

HEK-293T and MDCK.2 cells were purchased from American type culture collection (ATCC).
HeLa-ACE2/TMPRSS2 cells were from Dr. Jesse Bloom at the Fred Hutchinson Cancer Research Center and provided as a generous gift.
Expi-293F cells were purchased from Thermo Fisher.

Authentication

None of the cell lines were authenticated.

Mycoplasma contamination

All cell lines tested negative for Mycoplasma contamination.

Commonly misidentified lines
(See ICLAC register)

No commonly misidentified cell lines were used in the study.

Animals and other research organisms

Policy information about studies involving animals; ARRIVE guidelines recommended for reporting animal research, and Sex and Gender in Research

Laboratory animals

BALB/c mice (6-8 weeks) were purchased from Jackson Laboratories and randomly grouped for the studies.

Wild animals

No wild animals were used in the study.

Reporting on sex	Female BALB/c mice were used in the study.
Field-collected samples	No field-collected samples were used in the study.
Ethics oversight	All animals were maintained in accordance with the Public Health Service Policy for “Human Care and Use of Laboratory Animals” under a protocol approved by the Stanford University Administrative Panel on Laboratory Animal Care (APLAC-33709).

Note that full information on the approval of the study protocol must also be provided in the manuscript.

Flow Cytometry

Plots

Confirm that:

☒ The axis labels state the marker and fluorochrome used (e.g. CD4-FITC).

☒ The axis scales are clearly visible. Include numbers along axes only for bottom left plot of group (a 'group' is an analysis of identical markers).

☒ All plots are contour plots with outliers or pseudocolor plots.

☒ A numerical value for number of cells or percentage (with statistics) is provided.

Methodology

Sample preparation	To study the germinal center responses, we immunized BALB/c mice (n=10 mice per group) with 5 μg protein antigens (GP or GP-12D) adjuvanted with 150 μg alum via subcutaneous injection on days 0, 7 and 14 (two groups per time point). On day 21, all six groups of mice and another group of naïve mice were euthanized for tissue collection. Draining lymph nodes were collected and triturated through a 70-μm cell strainer to make single-cell suspensions, followed by staining for viability with Ghost Dye Violet 510 (Tonbo Biosciences).
Instrument	Data were acquired on a BD LSR-II flow cytometer.
Software	BD FACSDiva (version 8.0.1) was used for data collection on the BD LSR-II.<br>FlowJo (version 10.4.0) was used for data analysis.
Cell population abundance	No post-sorting fractions were collected.
Gating strategy	Cells were selected based on SSC-A vs. FSC-A and then singlets were selected based on FSC-H vs. FSC-A. Live CD19+CD3- cells were used for B cell analysis. Live CD19+CD3-CD95+CD38- B cells were gated for germinal center B cells. IgG+ germinal center B cells were selected based on SSC-H vs IgG. Live CD19-CD3+ cells were used for T cell analysis. Live CD19-CD3+CD4+PD1 +CXCR5+ T cells were gated for T follicular helper cells.

☒ Tick this box to confirm that a figure exemplifying the gating strategy is provided in the Supplementary Information.
