# Supplementary figures and images for: Vaccine design via antigen reorientation
Source: Nat Chem Biol. 2024 Jan 15;20(8):1012–21. doi: 10.1038/s41589-023-01529-6 (PMC11247139; doi:10.1038/s41589-023-01529-6)

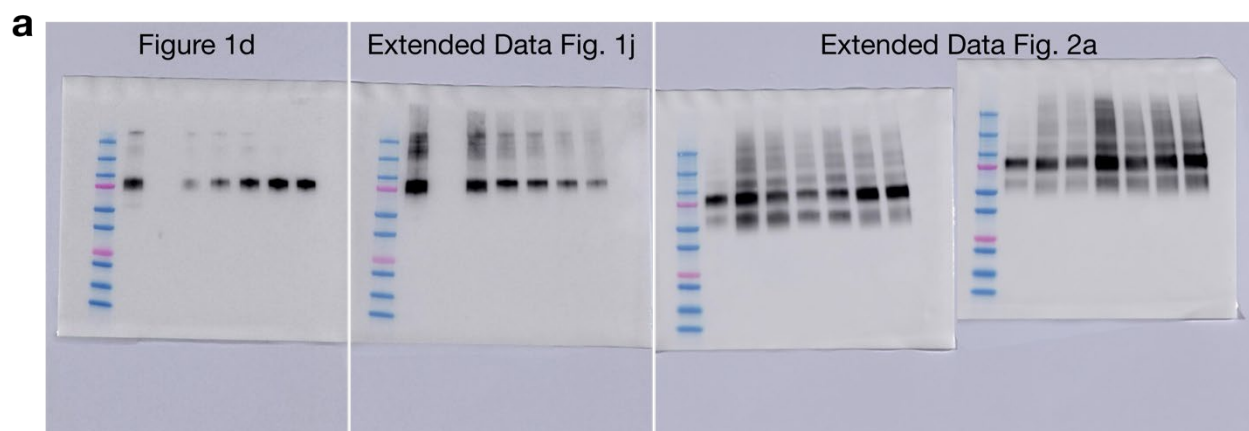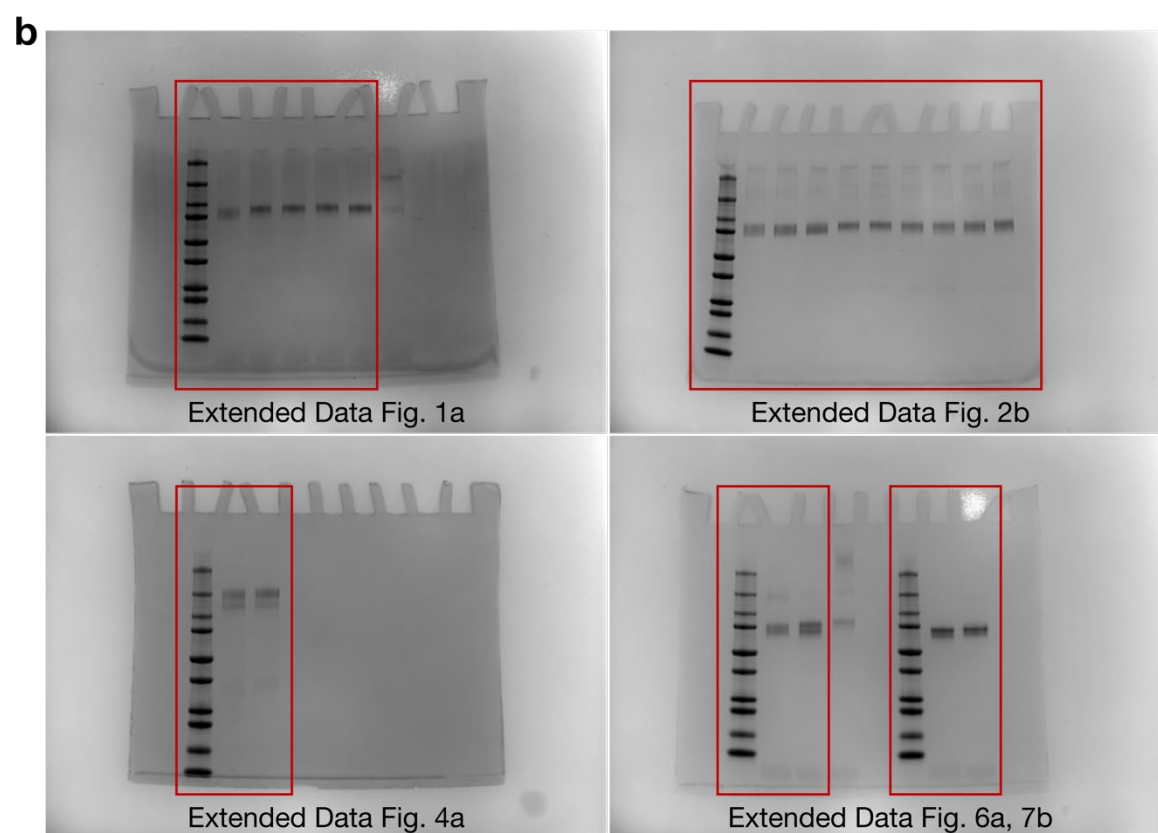

**Source data** | Uncropped scans of blots and gels in Figures and Extended Data Figures.

Supplement: Supplementary file 19 — Uncropped scans of blots and gels [file 41589_2023_1529_MOESM19_ESM.pdf]
